# Hydrochemical and isotopic baselines for understanding hydrological processes across Macquarie Island

**DOI:** 10.1038/s41598-022-25115-3

**Published:** 2022-12-08

**Authors:** Karina T. Meredith, Krystyna M. Saunders, Liza K. McDonough, Melodie McGeoch

**Affiliations:** 1 Securing Antarctica’s Environmental Future, The Australian Nuclear Science and Technology Organisation, New Illawarra Road, Lucas Heights, NSW 2234 Australia; 2 Institute for Marine and Antarctic Studies, University of Tasmania, Castray Esplanade, Battery Point, TAS 7004 Australia; 3 Securing Antarctica’s Environmental Future, Department of Environment and Genetics, La Trobe University, Melbourne, VIC 3086 Australia

**Keywords:** Biogeochemistry, Climate sciences, Ecology, Environmental sciences, Hydrology

## Abstract

Isotopic and hydrochemical data from lakes provide direct information on catchment response to changing rainfall, evaporation, nutrient cycling, and the health of ecosystems. These techniques have not been widely applied to lakes in the Southern Hemisphere high latitudes, including Southern Ocean Islands (SOIs) experiencing rapid, significant shifts in climate. Historical work has highlighted the localised nature of geochemical drivers in controlling the hydrochemical evolution of lakes, such as geology, sea spray contribution, vegetation, geographical location, and ice cover extent. The role of groundwater in lake hydrology and hydrochemistry has not been identified until now, and its omission will have major implications for interpreting soil–water–air processes affecting lakes. Here we present the first comprehensive, island-wide hydrochemical and isotopic survey of lakes on a SOI. Forty lakes were examined across Macquarie Island, using comparable methods to identify key environmental processes and their geochemical drivers. Methods include stable carbon (δ^13^C_DOC_: dissolved organic carbon and δ^13^C_DIC_: dissolved inorganic carbon), oxygen (δ^18^O), hydrogen (δ^2^H) and strontium isotopic ratios (^87^Sr/^86^Sr) in water. These provide essential baseline data for hydrological, biological, and geochemical lake processes. Lakes on the western side of the island are influenced by sea spray aerosols. In general, it was found that lakes at higher elevations are dilute and those located in lower elevation catchments have experienced more water–rock interactions. The hydrochemical and isotopic tracers suggest that lakes in lower elevations contain more terrestrial sourced ions that may be contributed from groundwater. Increasing temperatures and changing rainfall patterns predicted for the region will lead to shifts in nutrient cycles, and impact the island’s unique ecosystems. Future research will focus on long-term monitoring to understand seasonal, annual, and long-term variability to test fundamental hypotheses concerning ecosystem function and the consequences of environmental change on SOIs.

## Introduction

**Figure 1:**
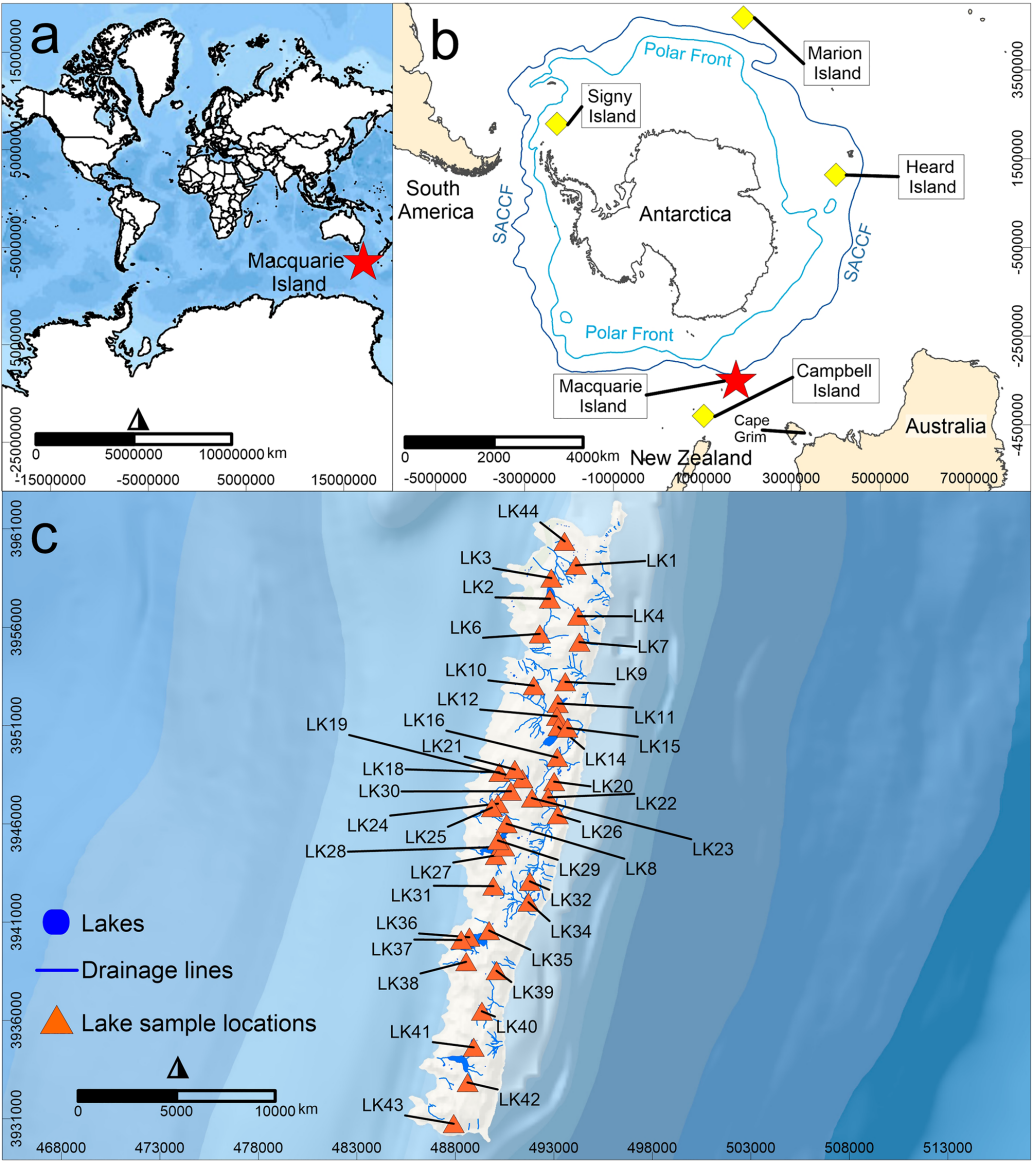
(**a**) The global context of the study area showing the location of Macquarie Island (indicated by a red star), map uses WGS84 coordinate system (**b**) the location of Macquarie Island (indicated by a red star) with respect to Antarctica, Australia and South America and the SOIs (Southern Ocean Islands), Polar Front and Southern Antarctic Circumpolar Current Front (SACCF), map uses the Polar Stereographic coordinate system, and (**c**) map of Macquarie Island with location of lake water sampling sites (orange symbols) with lake extents and drainage lines.

The application of isotopic tracers in water is essential for understanding lake hydrology and the fundamental processes driving lake water chemistry^1,2^. These techniques give us information on how catchments respond to rainfall, evaporation, groundwater influx and carbon and nutrient cycling^3–5^. Lake water can be used to provide a deeper understanding of past environment and ecosystem information contained within lake sediments to understand long-term environmental change for a region^6–8^. Investigating the hydrochemistry and hydrology of lakes in the Southern Hemisphere high latitudes, including the Southern Ocean Islands (SOIs) (Fig. 1a,b) that are experiencing rapid, significant shifts in climate and terrestrial ecosystems^9,10^ has not been widely undertaken. The only study to apply isotopic tracers to lake waters in the region used stable water isotopes (oxygen-18 and deuterium) in lakes across Signy Island (Fig. 1b). This study showed that lakes responded to seasonal change in rainfall and snowmelt, highlighting the importance of understanding the modern hydrology and hydrochemical characteristics of lakes for interpreting palaeoclimatic archives for SOIs^11^. While this study was limited to using only stable water isotopes, it demonstrated the enormous potential of using isotopic tracers for understanding island hydrology including identifying the sources of water to lakes. However, until now this approach has been neglected in other SOIs and is a missed opportunity to monitor changes in catchment hydrology.

While isotopes have not been used widely across the SOIs, lake water chemistry surveys have applied traditional hydrochemical and biological approaches that highlighted the localised nature of geochemical drivers controlling the hydrochemical evolution of lake waters such as geology, sea spray contribution, vegetation, geographical location, and ice cover extent^7,12–19^. The role of groundwater in lake hydrology and hydrochemistry has not been identified, and its omission could have major implications for interpreting soil–water–air processes affecting lakes, and ultimately influencing how palaeoclimate lake records are interpreted. Understanding how the hydrology of the SOIs will change due to a changing climate is critical for predicting catchment and ecosystem response to future changes in rainfall, evaporation, and groundwater exchange.

One particularly vulnerable island in the region, is Macquarie Island (Fig. 1c). It is small (124 km^2^), low-lying (maximum height of 433 m above sea level (a.s.l.) with low topographic variation, and is experiencing significant changes in multiple climate parameters such as rainfall^10^. As a result, the biodiversity of the island is under significant threat. For example, changes in water availability or hydrology of the island are suggested to be a primary cause of the widespread decline in the endemic, and now threatened, keystone cushion plant *Azorella macquariensis*^9,10,20^. The loss of the island’s iconic vegetation terracing is, as a result, also of concern.

The main purpose of this study is to focus on new hydrological findings by using a variety of isotopic techniques that have not yet been applied to SOIs. In this study, these techniques were used to understand the role of rainfall, sea spray aerosols (SSAs) and groundwater inputs in governing the lake water chemistry from 40 lakes across Macquarie Island. We test whether the isolation of Macquarie Island from large continental landmasses including the influence of the Southern Ocean is the dominant process driving the geochemistry of lakes across Macquarie Island. We also explore whether terrestrial processes such as the influx of terrestrially sourced ions from groundwater plays a significant role in the lake water chemistry. To do this we used sophisticated isotopic methods such as stable carbon including both dissolved organic carbon (δ^13^C_DOC_) and dissolved inorganic carbon (δ^13^C_DIC_), oxygen (δ^18^O), hydrogen (δ^2^H) and strontium isotopic ratios (^87^Sr/^86^Sr) to provide necessary information on geochemical processes that cannot be ascertained by only measuring the concentration of ions in surface water^3^. As such, it provides essential baseline data for understanding how changing climate variables such as rainfall and temperature for the region will impact the hydrological, biological, and geochemical processes in SOI lakes.

## Environmental setting

Macquarie Island (54°30′S, 158°57′E) is located in the Southern Ocean just north of the Polar Front (Fig. 1). It is 1500 km south-east of Tasmania, Australia, 1200 km southwest of New Zealand and 1500 km from the Antarctic continent. The island lies on the Australia/Indian and the Pacific tectonic plates, which are tectonically active^21^. It represents a rare example of uplifted oceanic crust that is part of the Macquarie Ridge Complex^22^ and emerged ca. 600,000 years ago^23^. Most of the island is composed of pillow basalt with faulting and dolerites. The north contains ultrabasic, gabbro and troctolites rock types^24^. Recent geological units include the palaeo-beach and lake deposits, alluvium, colluvium and peat, which occur along the coast^25^. The island has steep coastal slopes rising to a plateau 200–300 m a.s.l.. Faulting, uplift, sea-level changes, erosion and periglacial processes have shaped the surface and its lakes^26^. Widespread glaciation did not occur during the Last Glacial Maximum, although it is possible that perennial ice and snow accumulated in some areas^27^. Drainage features and extents of modern lake systems are shown in Fig. 1c. Lakes on the island have not been observed to freeze completely, but up to 10 cm of ice cover has occurred on small ponds^26^. The soils were found to be derived largely from the underlying igneous material^21^.

The Island has a cool oceanic climate with mean temperatures between 3.1 °C in winter to 6.6 °C in summer from 1948 to 2020^28^. The mean annual precipitation is 992 mm (1948–2020) with rainfall on ~ 316 days of the year. Due to almost constant cloud cover, light levels are generally low, with a mean annual average of 2.4 h of sunshine per day^28^. The island is vegetated at lower elevations with tussock grasslands, herbs and sedges, mosses, liverworts and lichens^10,26^ and there are no tall shrubs or trees^26^. All indigenous biota have colonised via long-distance dispersal^10^. Introduced vertebrates such as rabbits and rodents^29,30^ have had devastating impacts and led to unprecedented changes to the island^30^. However, since the successful eradication program which commenced in 2010 and was completed in 2014, the island has shown significant signs of recovery^31^.

## Methods

### Field sampling and major ion analyses

Lake waters were obtained from 40 lakes during the Austral summer of 2018 (Fig. 1c). Lake water sample locations were chosen based on spatial distribution across the island and logistical feasibility. Water samples were collected from 20 cm below the lake surface and field parameters (electrical conductivity (EC), oxidation–reduction potential, dissolved oxygen (DO), temperature, and pH) were measured. Water samples were collected and filtered through a 0.45 μm polyethersulphone high-capacity filter. Full details of the methodology for surface water sample collection are provided in Meredith et al.^3^. Anions and cations were analysed using inductively coupled plasma mass spectrometry (ICP-MS), inductively coupled plasma-atomic emission spectrometry (ICP-AES) and ion chromatography (IC) (Table S1).

### Environmental isotopes and dissolved organic carbon concentrations

The stable oxygen and hydrogen (δ^18^O and δ^2^H) isotopes were analysed by a Picarro L2130-i Cavity Ring-Down Spectrometer and reported as per mil (‰) deviations from the international standard V-SMOW with an analytical precision of ± 0.2 and ± 1.0‰, respectively. The stable carbon isotope values of dissolved inorganic carbon (δ^13^C_DIC_) values of waters were analysed by isotope ratio mass spectrometry (IRMS). After injecting the CO_2_ into a helium stream, which was separated from other gases by gas chromatography, it is attached to a Finnigan 252 mass spectrometer using a Conflo III. Results were reported as ‰ deviation from the international carbonate standard, NBS19 with a precision of ± 0.1‰. The dissolved organic carbon (DOC) and δ^13^C_DOC_ values were analysed using a total organic carbon analyser interfaced to a PDZ Europa20-20 IRMS utilising a GD-100 gas trap interface. Results were reported as ‰ deviation from the NIST standard reference material with an analytical precision of ± 0.6‰.

Strontium (Sr) isotopic ratios (^87^Sr/^86^Sr) from 10 of the lakes (LK2, LK6, LK14, LK29, LK31, LK34, LK37, LK38, LK43 and LK44) (Fig. 1c) were measured at the University of Melbourne using a Nu Plasma multi-collector ICPMS (MC-ICPMS) equipped with a CETAC Aridus desolvator and low-uptake Glass Expansion nebuliser (approximately 0.07 ml min^−1^). Approximately 20 g of water was evaporated in a HEPA-filtered fume hood. The Sr was then extracted using a single pass over a 0.15 millilitre column of EICHROM Sr resin^32^. Blanks were run alongside the samples with new resin used for each sample to eliminate memory issues. A 2% nitric acid was used to dissolve the dried Sr and obtain a concentration of 40–45 parts per billion. Krypton, rubidium, and strontium were corrected for cone memory and rubidium interference using gas blanks. Data are corrected for drift using standard SRM987. Any instrumental mass bias was removed by internal normalisation to ^88^Sr/^86^Sr = 8.37521 using the exponential law. Internal precision of the mass bias corrected ^87^Sr/^86^Sr is between 1.2 × 10^–5^ and 2.0 × 10^–5^. The estimated reproducibility (2σ) of standards and other materials on the multi-collector inductively coupled plasma mass spectrometer is estimated to be 4.0 × 10^–5^.

### Mapping and statistical analyses

Shapiro–Wilk tests for normality returned *p* values < 0.05, indicating that distributions of parameters are significantly different from normal. Statistical relationships between variables were therefore assessed using the non-parametric Spearman's rank correlation coefficient (⍴). A Geographical Information System (GIS) was developed in ArcMap 10.2.1 in the coordinate system WGS 1984 UTM zone 57S. Each parameter measured was added to the GIS to assess the spatial distribution of parameters across the island (Figs. S1–S11). Principal Component Analysis (PCA) was performed with parameters that were standardised by subtracting the mean and dividing by the standard deviation using the prcomp() function in R (v.1.1.456^33^). The following hydrochemical (in mmol l^−1^), isotopic (‰) and environmental parameters were used; distance to west coast (km), elevation (m), temperature (°C), DO (mg l^−1^), EC (µS cm^−1^), pH, Cl, SO_4_, SiO_2_, Na, Ca, K, Sr, Fe, Mn, δ^18^O, δ^2^H, δ^13^C_DIC_, DOC, δ^13^C_DOC_, F and Al. A hierarchical cluster analysis was used to group lakes based on their loadings on the PCA components using the HCPC() function (*FactoMineR* package^34^) in R (v.1.1.456^33^). To determine likely sources of Sr for each sample, a second PCA was performed to see which variables clustered with high and low ^87^Sr/^86^Sr values.

## Results

### Field parameters and major ions

The results of the hydrochemistry and environmental isotopes for the 40 lakes are presented spatially in Figs. S1–S11 and are located in Tables S1 and S2.

**Figure 2:**
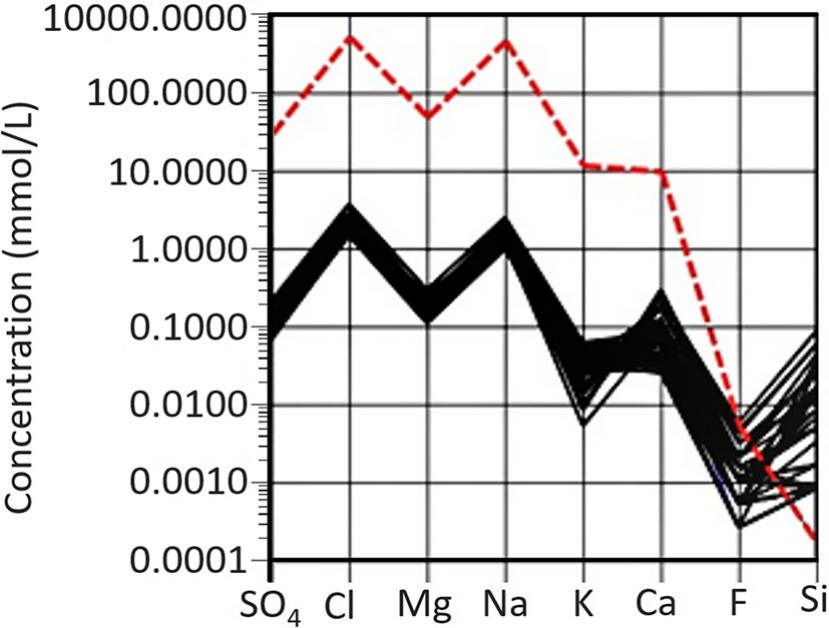
Lake waters sampled from Macquarie Island represented as a Scholler plot with the red dashed bolded line representing seawater composition and black lines are lake water concentration of SO_4_, Cl, Mg, Na, K, Ca, F and Si.

The lake waters are oxic (8.6–12.6 mg l^−1^) and range from slightly acidic (pH 6.0) to slightly alkaline (pH 9.2). Lake water temperatures are generally highest for lakes along the west coast (greater than 10 °C, Table S2). Phosphate concentrations are below detection level (0.1 mg l^−1^) for all lakes and nitrate was low ranging from below detection limit (< 0.05) to 0.21 mg l^−1^. A number of lakes have similar ionic ratios to seawater with Na–Cl type waters being the dominant cation and anion. Bicarbonate concentrations were calculated by difference and were low with a maximum of 36 mg l^−1^ and average of 3.7 mg l^−1^. All lakes are low in Cl concentrations ranging from 1.4 (LK22) to 3.7 (LK3) mmol l^−1^ and Na concentrations ranging from 0.99 (LK28) to 2.57 (LK3) mmol L^−1^. The SO_4_, Cl, Mg and Na concentrations follow a similar pattern to seawater in the Schoeller plot (Fig. 2), with all showing very high significant positive correlations (⍴ ≥ 0.75, *p* ≤ 1.4 × 10^–5^, Table S3). The K, Ca, F and Si concentrations follow a similar pattern to seawater for some lakes, whilst others diverge, with all lake waters containing higher Si concentrations than seawater (Fig. 2). An increase in Cl concentration is broadly reflected in the increase in Br, K and Sr (all ⍴ ≥ 0.71, *p* ≤ 1.7 × 10^–6^). The high correlation between these variables implies a similar source of ions, or that the waters have undergone similar hydrochemical processes. Some variables such as F and Cl, F and K, and Cl and Si, however, are not significantly correlated (⍴ = 0.24, − 0.26 and − 0.14, *p* = 0.1, 0.1 and 0.4 respectively, Table S3) suggesting they have a different source.

### Hierarchical cluster and principal component analysis

**Figure 3:**
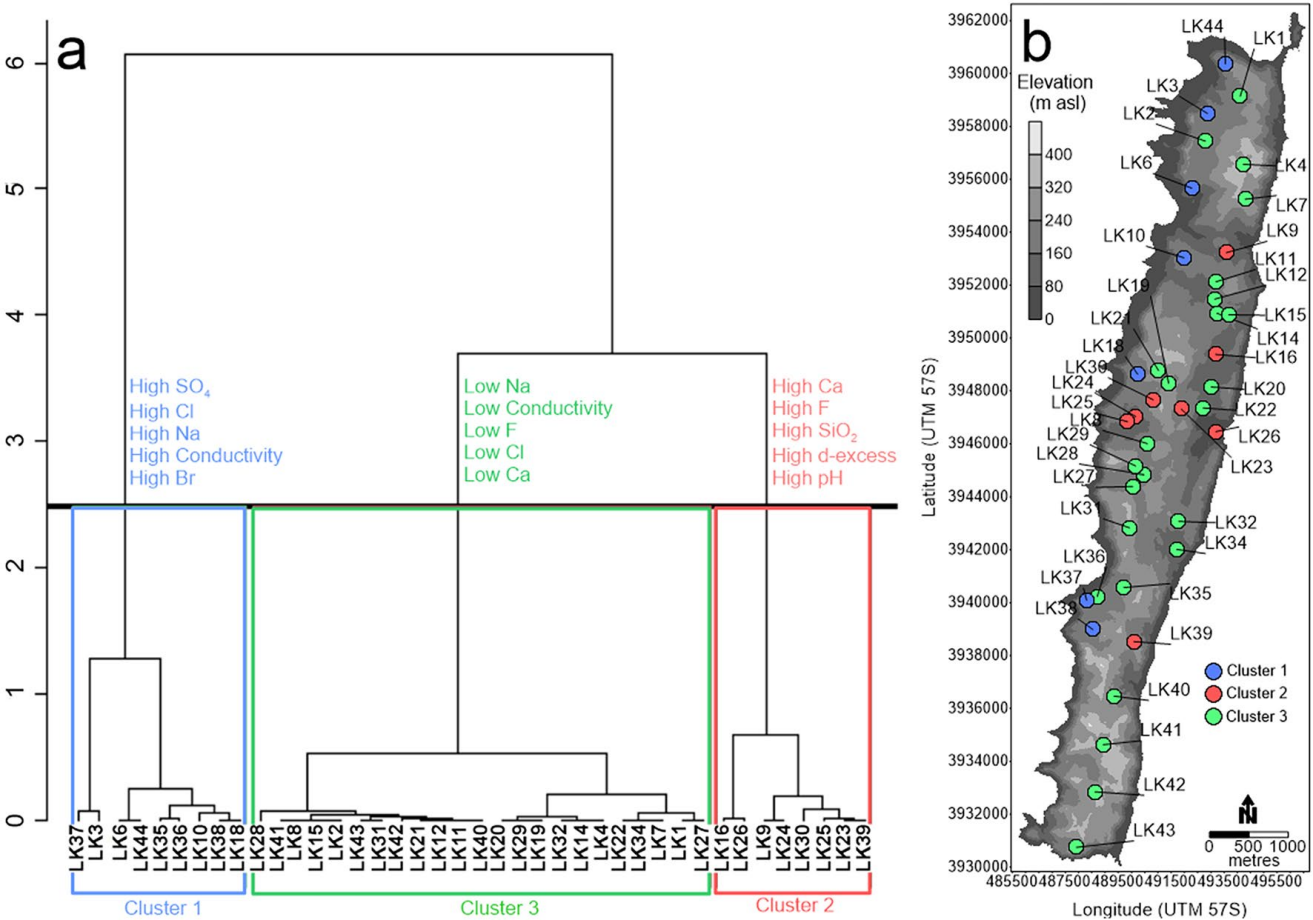
(**a**) Hierarchical cluster analysis of lake water chemistry variables on Macquarie Island, showing three lake clusters and the five most significant variables associated with each cluster (Table S4). (**b**) Lakes coloured by cluster membership superimposed on a layer of the islands elevational variation.

Hierarchical cluster analysis revealed three main clusters of lakes (Fig. 3). These clusters were used to colour code the results of the PCA to determine the main hydrochemical processes for the groupings. Cluster 1 represents lakes containing high SO_4_, Cl, Na, EC, Br, Sr, K, Mg, δ^2^H, Al and DOC concentration, and low pH, δ^13^C_DIC_ and smaller distance from the west coast (all *p* < 0.5, Table S4). Cluster 2 represents lakes containing high Ca, F, SiO_2_, d-excess, pH and Fe, low elevations, K and δ^2^H (all *p* < 0.5, Table S4). Cluster 3 represents lakes containing low Na, EC, F, Cl, Ca, SO_4_, Fe, Mg, DOC concentrations, Br, SiO_2_, Al and are located at higher elevations (all *p* < 0.5, Table S4).

PCA results show two main components explaining a total of 63% of the variability in the dataset. Components 1 and 2 of the PCA explain 41.9% and 21.1%, respectively. Variables loading most strongly on the first component (Dim1) include K, SO_4_, Sr, d-excess, Cl, δ^18^O, δ^2^H, Na and Br (Fig. S12). Variables loading most strongly on the second component (Dim2) include Ca, F, Fe, SiO_2_, DOC concentration, EC, elevation, Na and Mg (Fig. S12). Lakes on the western side of the island strongly influence Dim1 (i.e. LK3, LK6, LK10, LK23, LK30, LK37 and LK38) whilst lakes on the central and eastern portion of the island (i.e. LK11, LK12, LK21, LK24, LK40 and LK42) influenced Dim2 most strongly.

### Environmental isotopes and dissolved organic carbon concentrations

**Figure 4:**
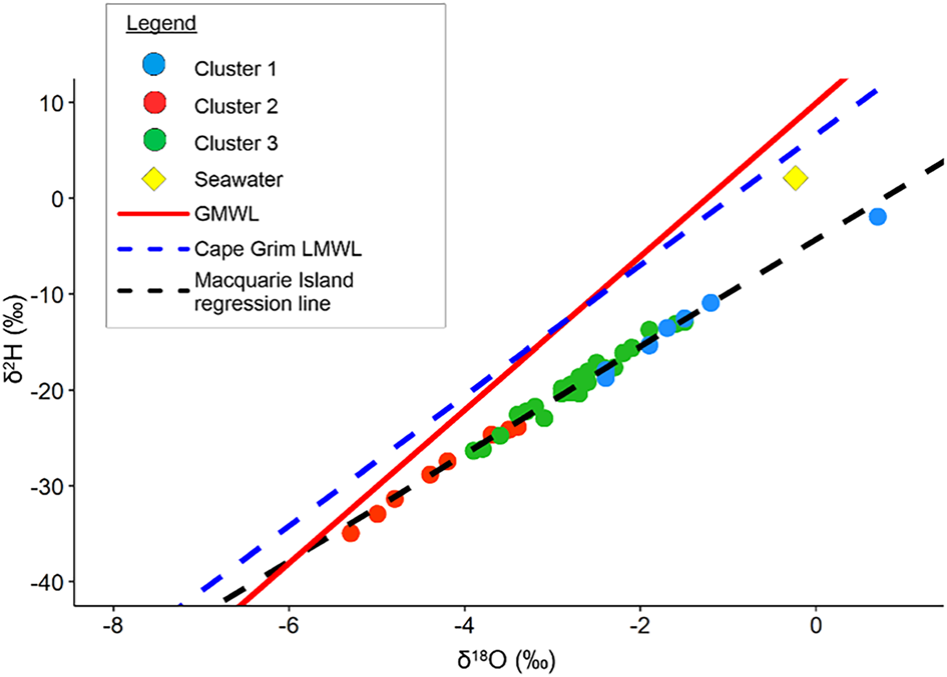
Difference between Macquarie Island Lake water samples (δ^2^H = 5.6 δ^18^O − 4.3), the global meteoric water line (GMWL, δ^2^H = 8 δ^18^O + 10) and Cape Grim local meteoric water line (LMWL, δ^2^H = 6.8 δ^18^O + 6.65). (Cluster groupings from Fig. 3a).

The δ^2^H and δ^18^O values range from − 34.9‰ (LK16) to − 1.9‰ (LK3) and − 5.30‰ (LK16) to + 0.70‰ (LK3), respectively (n = 40) (Table S2). Lake waters plot to the left of the Global Meteoric Water Line (GMWL, red solid line on Fig. 4) on a regression line described by δ^2^H = 8 δ^18^O + 10^35^, and the Cape Grim local meteoric water line (LMWL, δ^2^H = 6.8 δ^18^O + 6.65^36^ shown as a blue dashed line in Fig. 4). The average δ^2^H and δ^18^O values for the 40 lake waters is − 20.1‰ and − 2.83‰, respectively, which indicates they are slightly more enriched in ^18^O than the amount weighted rainfall values for Cape Grim (− 20.3‰ and − 3.97‰). The δ^18^O and δ^2^H values are not correlated with Cl (⍴ = 0.50 and 0.47, respectively) and do not plot on a mixing line towards seawater (shown as a yellow diamond in Fig. 4).

^87^Sr/^86^Sr ratios range from 0.70694 (LK29) to 0.70908 (LK38) with an average value of 0.708341 (n = 10). ^87^Sr/^86^Sr ratios were mildly positively correlated with Sr concentrations (⍴ = 0.64, *p* = 0.04) suggesting a possible source of Sr with high ^87^Sr/^86^Sr ratios. δ^13^C_DIC_ values for the lake waters are highly variable and range from − 23.1‰ (LK37) to − 1.5‰ (LK29) with an average of − 11.9‰ (n = 40). The DOC concentrations range from 0.7 (LK14) to 6.8 (LK44) mg l^−1^ with an average of 2.4 mg l^−1^ (n = 40). The δ^13^C_DOC_ values are more consistent and range from − 23.7‰ (LK35) to − 36.7‰ (LK19) with an average of − 28.2‰ (n = 40).

### Spatial variation in lake water chemistry

**Figure 5:**
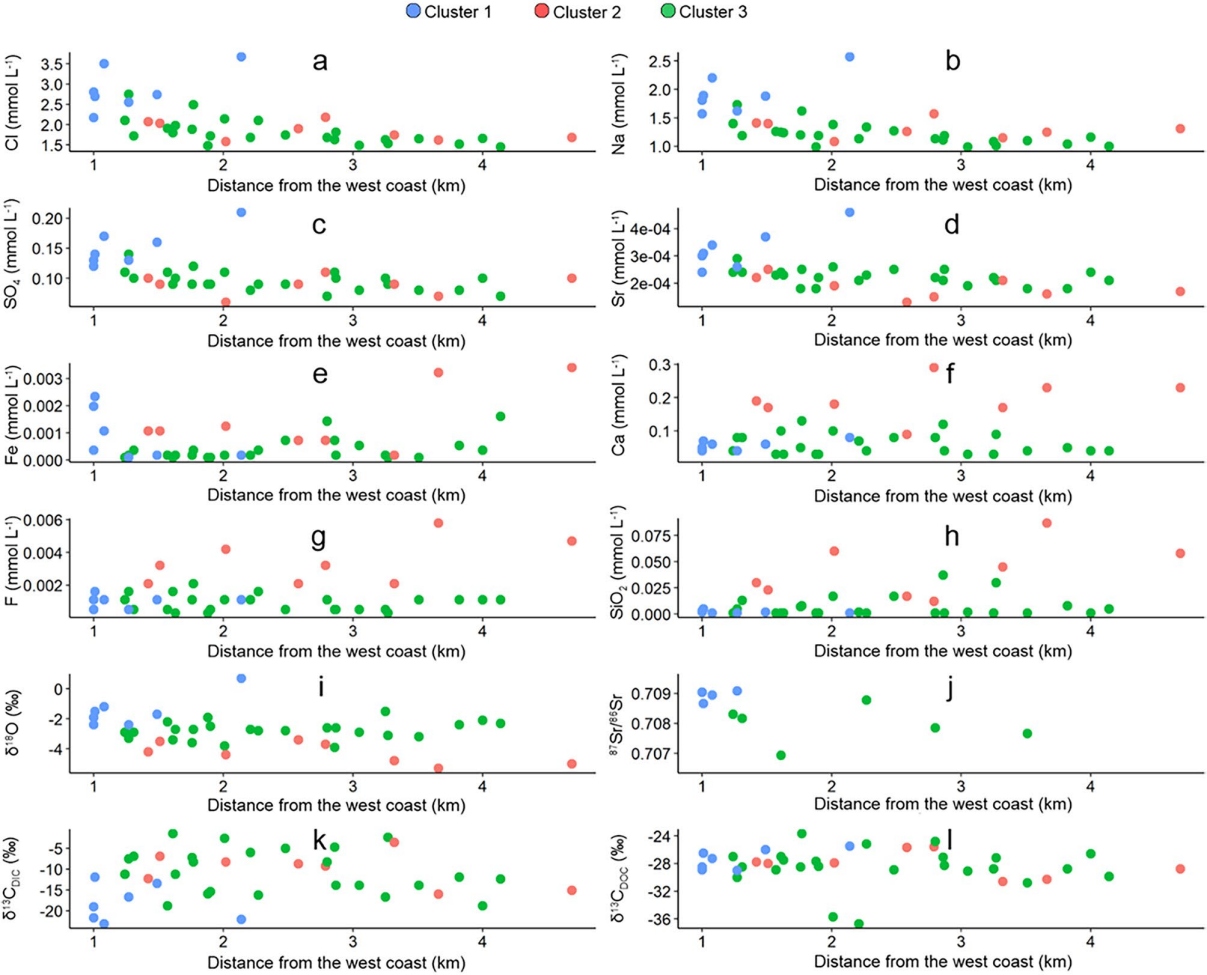
Distribution of ion concentrations and isotopes relative to distance from the west coast. Note the generally high concentrations of (**a**) Cl, (**b**) Na, (**c**) SO_4_ and (**d**) Sr in the lakes from Cluster 1 (green) are located close to the west coast. Concentrations of (**e**) Fe are mixed in lake waters impacted by sea-spray, whilst concentrations of (**f**) Ca, (**g**) F and (**h**) SiO_2_ are low in these lakes and increase with distance from the west coast, particularly in samples identified as having undergone high water–rock interaction. Lakes in Cluster 1 located close to the west coast contain higher (**i**) δ^18^O and (**j**) ^87^Sr/^86^Sr ratios and relatively low (**k**) δ^13^C_DIC_ values. δ^13^C_DOC_ values (**l**) show no significant relationship with distance from the west coast (*p* > 0.05).

The ionic concentration of the lake waters are controlled spatially, where Cl concentrations are highest along the west coast, and lowest in the centre, north and east coast of the island (Figs. S1 and 5a). Cl concentrations are not related to elevation (⍴ = 0.09, *p* = 0.6) or lake area (⍴ = 0.03, *p* = 0.8). The spatial distribution of K, SO_4_, Br, Na and Sr concentrations follows a similar pattern (Figs. S1–S3, Fig. 5b–d). Sr is significantly positively correlated with Cl (⍴ = 2.2 × 10^–7^), Br (⍴ = 1.5 × 10^–5^), Mg (⍴ = 7.2 × 10^–5^) and Na (⍴ = 2.6 × 10^–6^, Table S3). Overall, there is a general trend of decreasing Cl, Na, SO_4_ and Sr with increasing distance from the west coast, whilst elements such as Fe, Ca, F and Si and isotopes such as δ^18^O, δ^13^C_DIC_ and δ^13^C_DOC_ do not show a clear trend (Fig. 5e,h,i,k,l).

Sixteen of the lakes have SiO_2_ values of 0.001 mmol l^−1^, whilst LK16 contained the highest concentration (0.087 mmol l^−1^). LK16 is located at the second lowest elevation (at 95 m a.s.l) of lakes in this study (Table S2). Ca concentrations range from 0.02 mmol l^−1^ (LK8) to 0.29 mmol l^−1^ (LK9). LK8 is located at the third lowest elevation of lakes sampled in the study (Table S2). Three lakes have F concentrations of 0.0003 mmol l^−1^, whilst the highest F concentration is identified in LK16 at 0.0058 mmol l^−1^. SiO_2_, Ca and F are not significantly related to distance from the west coast (⍴ = 0.10, 0.22 and 0.08, *p* = 0.52, 0.18 and 0.61 respectively) (Fig. 5h,f,g) or Cl concentration (⍴ = 0.16, − 0.14 and 0.23, *p* = 0.33, 0.37 and 0.15 respectively). Fe and F are, however, significantly negatively correlated with elevation (⍴ = − 0.49, *p* = 1.5 × 10^–3^ and ⍴ = -0.41, *p* = 9.4 × 10^–3^ respectively). Ca and F are positively correlated with SiO_2_ (⍴ = 0.78 and 0.49, *p* = 2.4 × 10^–9^ and 1.3 × 10^–3^ respectively, Table S3) suggesting that their source is different from that of SO_4_, Cl, Mg and K (see also Fig. 2).

## Discussion

The following discussion provides new hydrological findings for lakes across Macquarie Island based on hydrochemical and isotopic measurements. Macquarie Island is a remote field location, and the rainfall has not been sampled or measured for isotopes or hydrochemistry, and neither has the physical hydrology or hydrogeology of the aquifers been investigated. Inference of the rainfall isotope composition for Macquarie Island is made based on global data. This is because the nearest high-resolution rainfall and aerosol collection site is located at Cape Grim on the north west coast of Tasmania^37^ (Fig. 1b), which is approximately 1800 km north west of Macquarie Island. Considering these limitations, the lake hydrochemistry of Macquarie Island is explored in the following discussion.

### Hydrological findings

**Figure 6:**
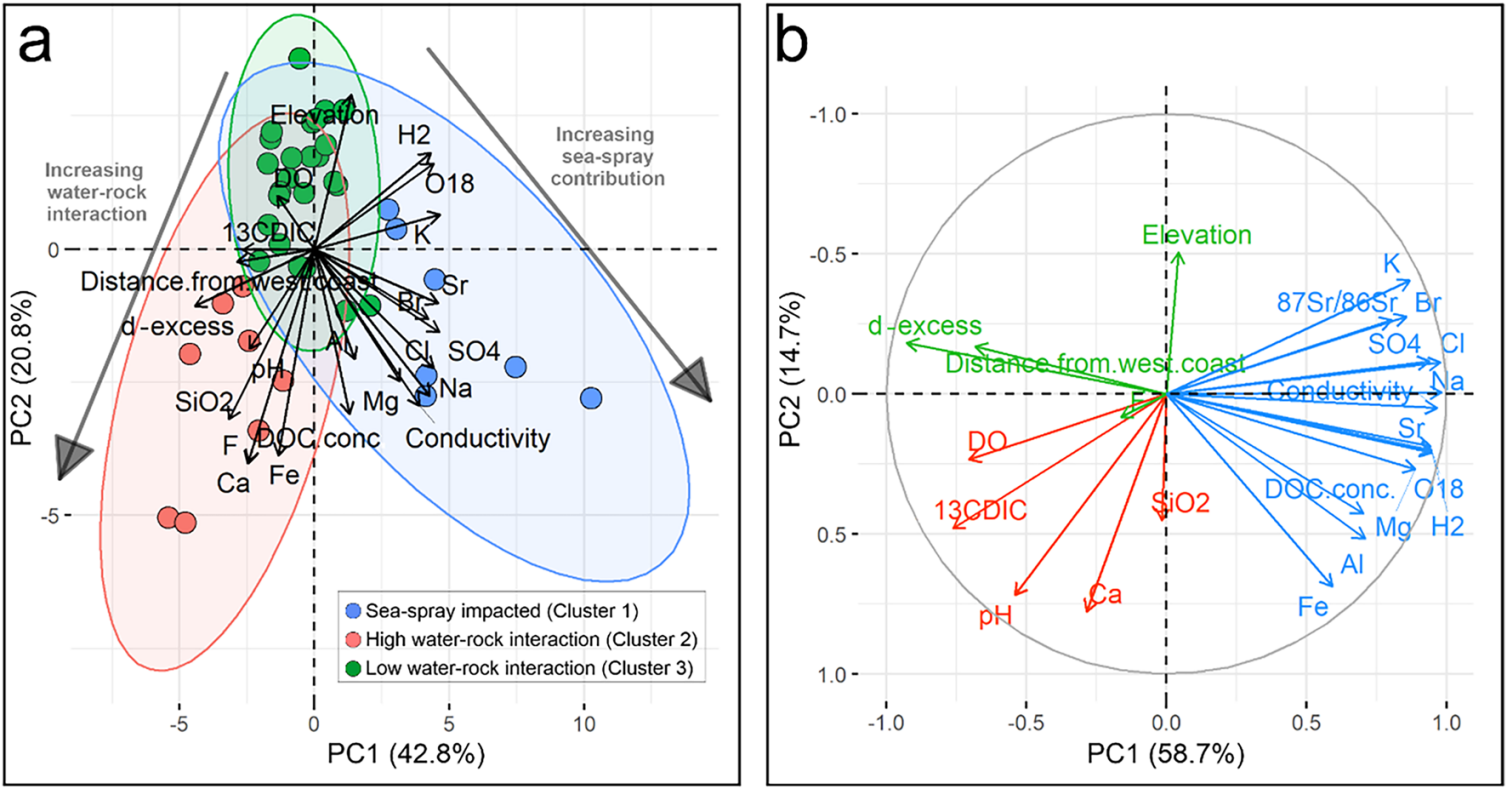
(**a**) Principal Component Analysis (PCA) of Macquarie Island lakes excluding ^87^Sr/^86^Sr. ^87^Sr/^86^Sr results were omitted from this PCA due to the low sample size (n = 10), which would result in the omission of 30 out of 40 lakes in the dataset and a reduction of the statistical power of the PCA. Sample colours represent lake clusters (see Fig. 3). Clusters are interpreted as groups of lakes impacted by sea-spray (Cluster 1 in Fig. 3), and two clusters less impacted by sea-spray, but impacted by high and lower water–rock interactions (Clusters 2 and 3 respectively in Fig. 3). (**b**) PCA of Macquarie Island lakes, including ^87^Sr/^86^Sr (n = 10) results. This biplot shows that the highest ^87^Sr/^86^Sr values in the dataset are associated with sea-spray inputs including SO_4_, Br, Cl, Na and conductivity. In contrast, low ^87^Sr/^86^Sr values are associated with lake waters which have undergone high water–rock interaction and the dissolution of secondary carbonates resulting in high ^13^C_DIC_, pH, Ca and HCO_3_. Note: The Component 2 (PC2) axis in (**b**) is flipped for easy comparison to (a).

The underlying geology and its structures control surface water and groundwater expressions across the island. Lakes across the island have not been observed to freeze completely, but ice cover can occur on small ponds. The lakes range from closed to open systems and do not show evidence of overflow. Groundwater springs have not been mapped across the island, but the elevational changes and Landsat images suggest surface expressions related to groundwater discharge sites. The δ^2^H and δ^18^O values of lake waters do not suggest a snowmelt origin as found across the Signy Island^11^ or a seawater origin, despite a strong SSA input (Fig. 4). Instead, lakes were slightly more enriched in δ^18^O (by over 1‰) but had similar δ^2^H values compared to the amount weighted rainfall values observed at Cape Grim^36^ (Fig. 4). However, this enrichment trend is observational and needs to be tested with further studies because Cape Grim is located ~ 1800 km northwest of Macquarie Island, and the localised rainfall patterns may differ greatly. The higher δ^18^O values suggest evaporation of the lakes, which may be related to the area of the lake, suggesting smaller lakes are experiencing more evaporation. These findings show that the major source of water to lakes across Macquarie Island is from a rainfall source that may be from direct precipitation into the lake catchment or by rainfall that has been recharged as groundwater into the underlying bedrock aquifer. The lakes can be broadly grouped into three clusters based on hydrochemistry and isotopic compositions (Fig. 6).

**Figure 7:**
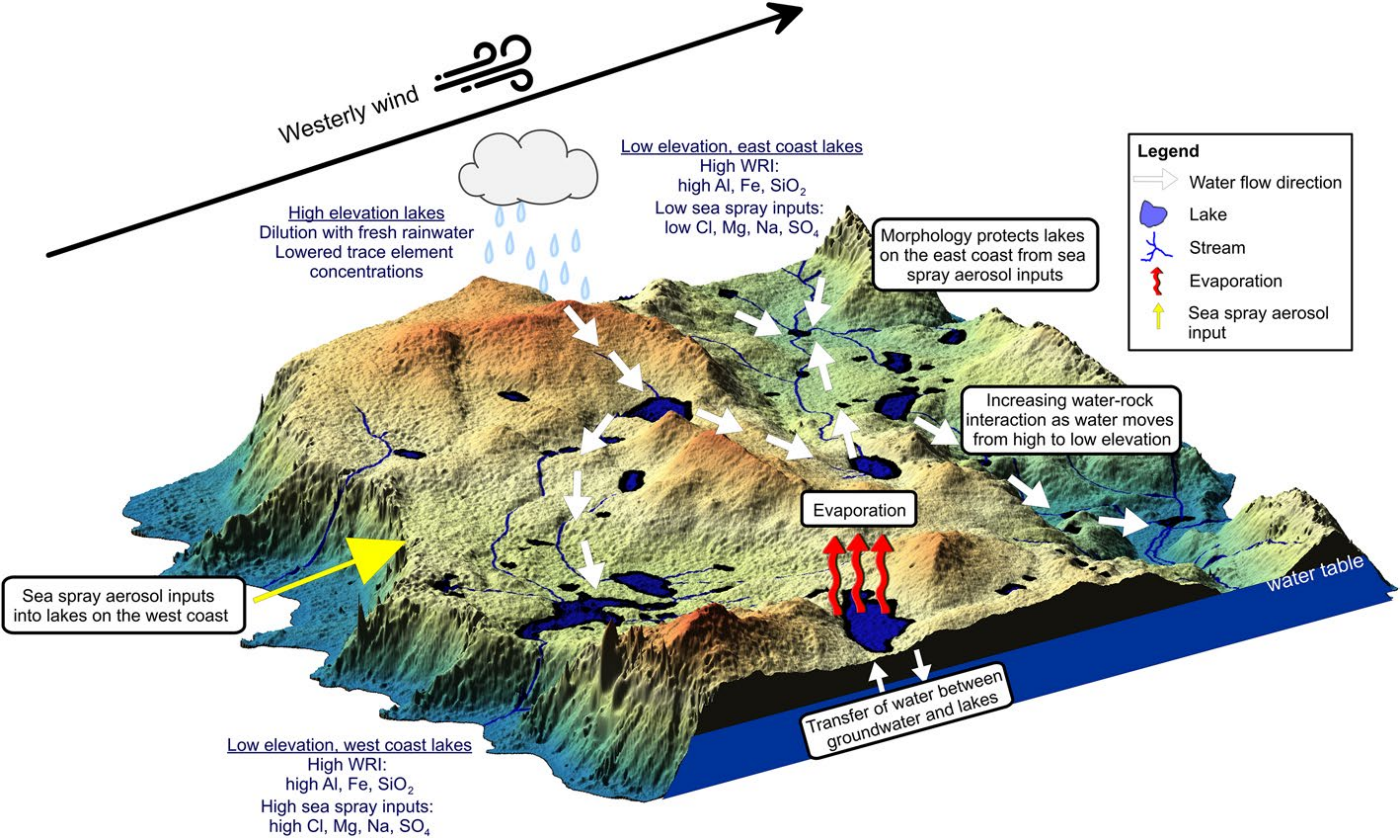
Conceptual synthesis of the hydrochemical processes affecting lake water chemistry on Macquarie Island.

Cluster 1 is associated with lakes on the western side of the island and while dilute in concentration compared to seawater, they contain higher concentrations of Na, Cl, Mg, K and SO_4_, ions which are found in seawater^38,39^. The highest concentrations of these ions occur along the west coast (LK3, 6, 10, 18, 37, 38, 44) and decrease in concentration with increasing distance from the west coast (Fig. 7). Within Cluster 1, four samples (LK6, 37, 38, 44) were analysed for ^87^Sr/^86^Sr isotope ratios (0.70866, 0.70894, 0.70908, 0.70904, respectively) and were close to the isotopic ratio of seawater ^87^Sr/^86^Sr (0.709, Fig. 5j). LK6 had a slightly lower value suggesting a lighter source of Sr for this lake.

Between 370 and 2423 kg per hectare per year of NaCl is deposited over Macquarie Island ^40^. The lakes accumulate salt over time, with concentrations likely to be dependent on the distance to the ocean, dilution from rainfall and evaporation. SSA contributions to lakesalong the west coast, match previous lake investigations that found that the main environmental process influencing lake water chemistry is the input of SSA; controlled by the prevailing westerly winds and the distance of the lake from the ocean on the western side of Macquarie Island^7,12–16^, however this process does not explain the hydrochemical variations observed for lakes in Clusters 2 and 3.

The PCA analysis also explains terrestrial processes associated with water–rock interaction and they are associated with elevation. Lakes increase in Ca, F and Si concentration with distance from the west coast (Fig. 5f,g,h). Cluster 2 has lakes located at lower elevations and are dominated by terrestrially sourced ions (LK9, 16, 23, 24, 25, 26, 30, 39) (Table S4, Fig. 4).

Cluster 3 contains lakes located at higher elevations (~ 150–300 m asl) with terrestrially sourced solutes but at lower concentrations than Cluster 2. Dissolved organic carbon concentrations are lower in the higher elevations but the δ^13^C_DOC_ values were generally close to soil organic matter values (− 24.7 to − 29.2‰^21^) and within the freshwater dissolved organic carbon, particulate organic carbon and algae range^41^. The similarity between soil organic matter and DOC values in lake waters suggests limited in situ biological processing in the lakes.

**Figure 8:**
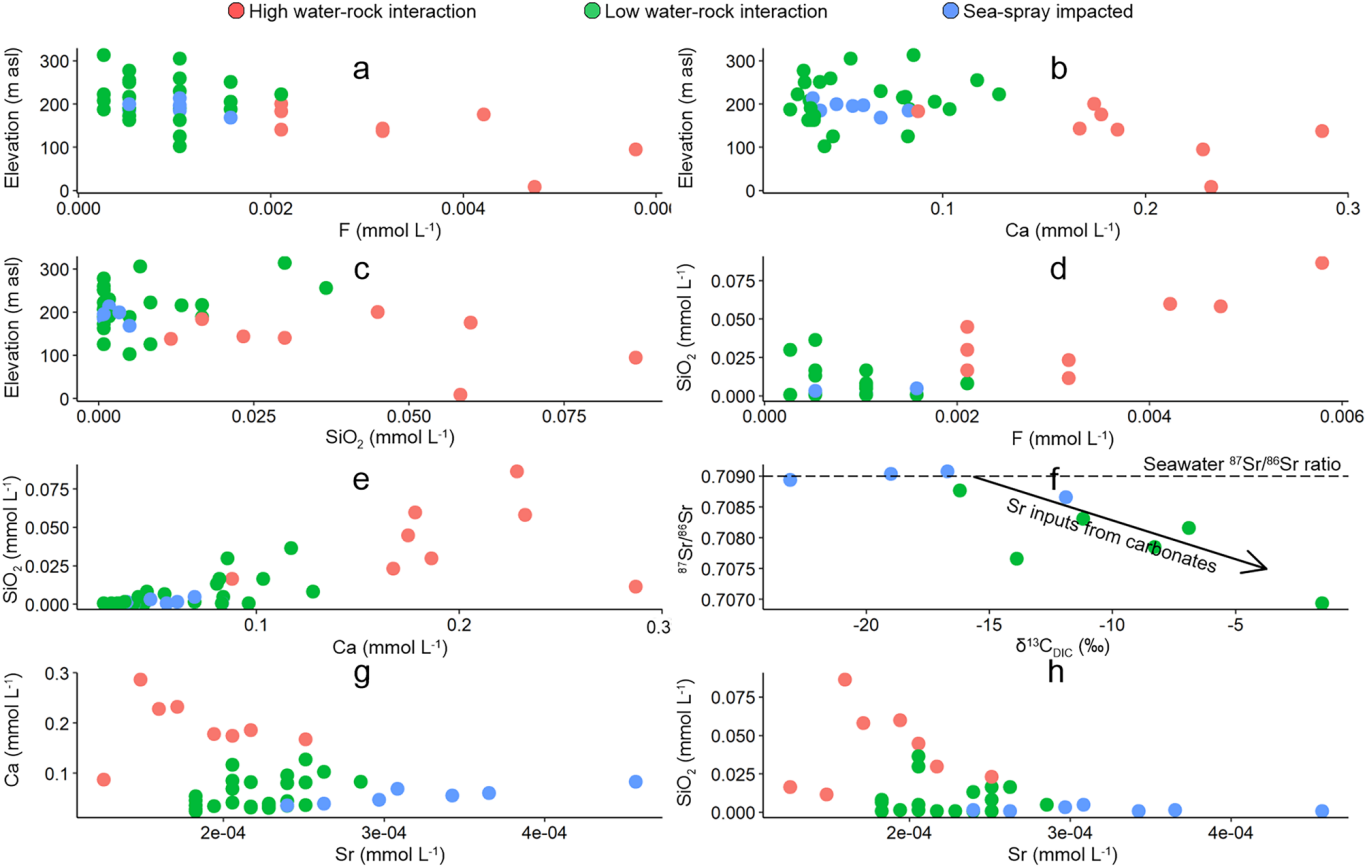
Relationships between (**a**) fluoride (F) vs. elevation, (**b**) calcium (Ca) vs. elevation, (**c**) silicon dioxide (SiO_2_) vs. elevation, (**d**) F vs. SiO_2_, (**e**) Ca vs. SiO_2_, (**f**) δ^13^C_DIC_ (‰) vs. strontium isotope ratios (^87^Sr/^86^Sr), (**g**) Sr vs. Ca and (**h**) Sr vs. SiO_2_. Lakes are coloured by cluster with red, green and blue points representing lakes with high sea-spray input and samples which have undergone high and low water–rock interaction (cluster groups 1, 2 and 3 respectively in Fig. 3a). Notably, the highest ^87^Sr/^86^Sr values (n = 10) in the dataset are associated with sea-spray inputs including SO_4_, Br, Cl, Na and conductivity.

Terrestrially derived solutes are most likely to be from the weathering of the underlying volcanic bedrock, which is predominantly basalt^24^. Lower elevation lakes have higher concentrations of terrestrially sourced solutes such as F, Ca and Si (Fig. 8a–c) due to increasing water–rock interaction (Fig. 8d,e). The highest Fe concentrations were associated with terrestrial sourced samples (Cluster 2) at the lowest elevations (< 100 m a.s.l.). This suggests that more dilute lakes at higher elevations are rainfall fed where waters from Cluster 2 and 3 sit closer to the GMWL (Fig. 4). Higher salinity waters containing terrestrial solutes located at the lower elevations are likely to be from either the flow of rainfall across the landscape during larger rainfall events resulting in water flowing from higher to lower elevations within localised lake catchments, or from groundwater influx from the underlying bedrock aquifer where the water has undergone greater water–rock interaction processes (Fig. 7). The later is a more likely explanation given that groundwater input can represent up to half of the annual water inputs and in some cases can be the primary source of ions into closed lake systems^42^.

^87^Sr/^86^Sr ratios can be used to trace groundwater^43^ and can be particularly useful in the identification of Sr from bedrock sources (silicate weathering versus carbonate weathering^5^) due to their different isotopic ratios. The decay of ^87^Rb into ^87^Sr, which is incorporated into silicate minerals due to the substitution of Rb in K-bearing minerals, results in high ^87^Sr/^86^Sr ratios in waters that have interacted with silicates (up to 0.7625 in silicates^44^) compared to seawater. In contrast, young oceanic basalts, such as those comprising Macquarie Island, contain low ^87^Sr/^86^Sr ratios (approximately 0.702–0.705) due to their low Rb/Sr ratios and lower radioactive ^87^Rb decay compared to older rocks^45^. The ^87^Sr/^86^Sr ratios of lake waters from Cluster 3 range from 0.70694 (LK29) to 0.70877 (LK43), which is slightly higher than the bedrock value. LK29 with the lowest ^87^Sr/^86^Sr ratio also has an enriched δ^13^C_DIC_ value (− 1.5‰), which likely indicates carbonate dissolution. The negative relationship between δ^13^C_DIC_ and ^87^Sr/^86^Sr suggests dissolution of carbonates associated with basalts (Fig. 8f). This may be in the form of carbonate veins, which have previously been identified in Macquarie Island peridotites^46^. This trend is supported by the relationship between Sr, Ca (Fig. 8g) and Si (Fig. 8h). However, δ^13^C_DIC_ values for LK14 (− 13.9‰) and LK31 (− 11.2‰) do suggest dissolved inorganic carbon from a groundwater source, even though ^87^Sr/^86^Sr ratios are much higher than the oceanic basalt values of 0.702–0.705. Whether the Sr is sourced from carbonate contained in the bedrock or the bedrock itself, these findings are significant showing that these lake waters likely have groundwater inputs.

### Implications

Baseline isotopic and hydrochemical values of lakes across Macquarie Island presented in this study provide fundamental information for further studies to focus on quantifying how changing rainfall patterns driven by climate change may affect lake water composition, and evaporation. This is particularly important given that current climate projections under a range of 1.5–4 °C global warming scenarios suggest temperature increases of approximately 1.5 °C–2 °C and precipitation increases of approximately 10%–60% at Macquarie Island^47^. Episodic increases in the proportion of lake water coming from precipitation during extreme events will likely result in periods of high rainfall leading to strong dilution and greater solute concentration ranges in higher elevation lakes. In contrast, increased groundwater table height in the underlying aquifer because of increased precipitation may increase terrestrially derived ions from groundwater inputs or overland flow in lower elevation lakes. Previous studies have identified that increased sunshine hours and a change in rainfall patterns are leading to large, episodic rainfall events, resulting in a reduction in plant water availability^9,10,20^ and likely shifts in lake water nutrient concentrations due to changes in vegetation cover^20^. Macquarie Island hosts unique ecosystems and with warming temperatures and changes in rainfall patterns this will impact the hydrology of lake catchments. The transportation of nutrients via overland flow processes will influence the growth and distribution of unique vegetation. Understanding the sources of water and the processes affecting current lake water chemistry, and how these may change in a warming climate, provide insights into potential impacts on the ecology of the island and its lakes due to climate change.

## Conclusion

This study is the first to show that terrestrial sourced ions most likely from groundwater is a major contributor to lakes across Macquarie Island. Lakes on the western side of the island are primarily influenced by SSAs. Lakes at higher elevations are generally dilute and those located in lower catchments have experienced more water–rock interactions as water moves from higher to lower elevations. We are unable to conclusively prove whether the water comes from overland flow, groundwater or sub-surface flow with this dataset. Future hydrological research on Macquarie Island and other SOIs will focus on long-term monitoring of lake and rainfall to understand seasonal, annual, and long-term variability and change. This research is required urgently because lake systems have unique environments and ecosystems that are facing unprecedented pressure from climate change. Long-term multi-disciplinary research including baseline surveys are needed to test fundamental hypotheses concerning ecosystem function and nutrient cycling on Macquarie Island and other SOIs.

## Supplementary Information

Supplementary Information.

## Data Availability

The full dataset used in this study is available upon request to the corresponding author.

## Code availability

The code used to develop individual figures is available upon request to the corresponding author.
