# Supplementary material for: Hydrochemical and isotopic baselines for understanding hydrological processes across Macquarie Island
Source: Sci Rep. 2022 Dec 8;12:21266. doi: 10.1038/s41598-022-25115-3 (PMC9732353; doi:10.1038/s41598-022-25115-3)
Supplement: Supplementary file 1 — Supplementary Information. [file 41598_2022_25115_MOESM1_ESM.docx]

Supplementary Information for:

# Hydrochemical and isotopic baselines for understanding hydrological processes across Macquarie Island

Karina. T. Meredith^1^, Krystyna. M. Saunders^1,2^, Liza. K. McDonough^1^, Melodie McGeoch^3^.

1. Securing Antarctica’s Environmental Future, The Australian Nuclear Science and Technology Organisation, New Illawarra Road, Lucas Heights, NSW, 2234, Australia
2. Institute for Marine and Antarctic Studies, University of Tasmania, Castray Esplanade, Battery Point, TAS, 7004, Australia
3. Securing Antarctica's Environmental Future, Department of Environment and Genetics, la Trobe University, Melbourne 3086, Vic. Australia.

*Correspondence to*: Karina. T. Meredith (kmj@ansto.gov.au)

**Table S1. Anion and cation results (mmol L^-1^).**

| Sample ID | Cl | SO_4_ | Br | SiO_2_ | Na | Ca | Mg | K | Sr | Fe | Mn | F | Al |
| --- | --- | --- | --- | --- | --- | --- | --- | --- | --- | --- | --- | --- | --- |
| LK1 | 1.7 | 0.09 | 0.001 | 0.017 | 1.27 | 0.08 | 0.20 | 0.034 | 0.00025 | 0.00072 | 0.00002 | 0.00053 | 0.00371 |
| LK2 | 1.7 | 0.10 | 0.002 | 0.013 | 1.19 | 0.08 | 0.19 | 0.033 | 0.00024 | 0.00036 | 0.00001 | 0.00053 | 0.00074 |
| LK3 | 3.7 | 0.21 | 0.005 | 0.001 | 2.57 | 0.08 | 0.30 | 0.064 | 0.00046 | 0.00018 | 0.00005 | 0.00105 | 0.00148 |
| LK4 | 1.5 | 0.09 | 0.002 | 0.030 | 1.01 | 0.09 | 0.17 | 0.029 | 0.00021 | 0.00009 | 0.00001 | 0.00026 | 0.00111 |
| LK6 | 2.7 | 0.14 | 0.004 | 0.005 | 1.89 | 0.07 | 0.21 | 0.043 | 0.00031 | 0.00233 | 0.00005 | 0.00158 | 0.00593 |
| LK7 | 1.6 | 0.11 | 0.002 | 0.037 | 1.11 | 0.12 | 0.19 | 0.023 | 0.00021 | 0.00072 | 0.00002 | 0.00053 | 0.00185 |
| LK8 | 1.6 | 0.09 | 0.002 | 0.001 | 1.06 | 0.02 | 0.12 | 0.032 | 0.00018 | 0.00018 | 0.00007 | 0.00026 | 0.00111 |
| LK9 | 2.2 | 0.11 | 0.002 | 0.012 | 1.57 | 0.29 | 0.26 | 0.008 | 0.00015 | 0.00072 | 0.00001 | 0.00316 | 0.00037 |
| LK10 | 2.7 | 0.16 | 0.003 | 0.002 | 1.88 | 0.06 | 0.21 | 0.051 | 0.00037 | 0.00018 | 0.00002 | 0.00105 | 0.00185 |
| LK11 | 1.8 | 0.10 | 0.003 | 0.001 | 1.19 | 0.04 | 0.14 | 0.034 | 0.00025 | 0.00018 | 0.00005 | 0.00053 | 0.00074 |
| LK12 | 1.6 | 0.10 | 0.002 | 0.001 | 1.08 | 0.03 | 0.12 | 0.029 | 0.00022 | 0.00018 | 0.00004 | 0.00053 | 0.00074 |
| LK14 | 1.6 | 0.08 | 0.002 | 0.001 | 1.10 | 0.04 | 0.13 | 0.031 | 0.00018 | 0.00009 | 0.00001 | 0.00105 | 0.00037 |
| LK15 | 1.7 | 0.10 | 0.002 | 0.001 | 1.16 | 0.04 | 0.13 | 0.031 | 0.00024 | 0.00036 | 0.00004 | 0.00105 | 0.00148 |
| LK16 | 1.6 | 0.07 | 0.002 | 0.087 | 1.25 | 0.23 | 0.13 | 0.015 | 0.00016 | 0.00322 | 0.00015 | 0.00579 | 0.00074 |
| LK18 | 2.2 | 0.13 | 0.004 | 0.002 | 1.57 | 0.04 | 0.13 | 0.049 | 0.00024 | 0.00036 | 0.00004 | 0.00105 | 0.00185 |
| LK19 | 1.7 | 0.08 | 0.002 | 0.002 | 1.13 | 0.07 | 0.15 | 0.030 | 0.00021 | 0.00018 | 0.00001 | 0.00105 | 0.00111 |
| LK20 | 1.5 | 0.08 | 0.002 | 0.008 | 1.04 | 0.05 | 0.14 | 0.030 | 0.00018 | 0.00054 | 0.00005 | 0.00105 | 0.00148 |
| LK21 | 1.7 | 0.09 | 0.002 | 0.001 | 1.19 | 0.03 | 0.14 | 0.034 | 0.00022 | 0.00009 | 0.00002 | 0.00053 | 0.00037 |
| LK22 | 1.4 | 0.07 | 0.002 | 0.005 | 1.00 | 0.04 | 0.12 | 0.024 | 0.00021 | 0.00161 | 0.00015 | 0.00105 | 0.00259 |
| LK23 | 1.7 | 0.09 | 0.002 | 0.045 | 1.15 | 0.17 | 0.16 | 0.032 | 0.00021 | 0.00018 | 0.00001 | 0.00211 | 0.00074 |
| LK24 | 2.0 | 0.09 | 0.002 | 0.023 | 1.40 | 0.17 | 0.18 | 0.023 | 0.00025 | 0.00107 | 0.00005 | 0.00316 | 0.00334 |
| LK25 | 2.1 | 0.10 | 0.002 | 0.030 | 1.41 | 0.19 | 0.15 | 0.013 | 0.00022 | 0.00107 | 0.00005 | 0.00211 | 0.00148 |
| LK26 | 1.7 | 0.10 | 0.002 | 0.058 | 1.31 | 0.23 | 0.11 | 0.010 | 0.00017 | 0.00340 | 0.00027 | 0.00474 | 0.00148 |
| LK27 | 2.1 | 0.11 | 0.003 | 0.017 | 1.38 | 0.10 | 0.17 | 0.035 | 0.00026 | 0.00018 | 0.00004 | 0.00105 | 0.00074 |
| LK28 | 1.5 | 0.09 | 0.002 | 0.001 | 0.99 | 0.03 | 0.11 | 0.026 | 0.00018 | 0.00009 | 0.00002 | 0.00026 | 0.00037 |
| LK29 | 1.8 | 0.09 | 0.002 | 0.001 | 1.25 | 0.10 | 0.15 | 0.031 | 0.00024 | 0.00009 | 0.00001 | 0.00158 | 0.00074 |
| LK30 | 1.6 | 0.06 | 0.001 | 0.060 | 1.08 | 0.18 | 0.11 | 0.010 | 0.00019 | 0.00125 | 0.00005 | 0.00421 | 0.00185 |
| LK31 | 2.1 | 0.11 | 0.003 | 0.001 | 1.40 | 0.04 | 0.16 | 0.043 | 0.00024 | 0.00009 | 0.00002 | 0.00105 | 0.00074 |
| LK32 | 1.5 | 0.08 | 0.002 | 0.002 | 0.99 | 0.03 | 0.11 | 0.025 | 0.00019 | 0.00054 | 0.00004 | 0.00053 | 0.00259 |
| LK34 | 1.7 | 0.07 | 0.002 | 0.001 | 1.13 | 0.08 | 0.14 | 0.019 | 0.00022 | 0.00143 | 0.00002 | 0.00105 | 0.00111 |
| LK35 | 2.5 | 0.12 | 0.003 | 0.008 | 1.62 | 0.13 | 0.20 | 0.044 | 0.00025 | 0.00036 | 0.00002 | 0.00211 | 0.00185 |
| LK36 | 2.8 | 0.14 | 0.004 | 0.005 | 1.73 | 0.08 | 0.21 | 0.045 | 0.00029 | 0.00018 | 0.00004 | 0.00158 | 0.00037 |
| LK37 | 3.5 | 0.17 | 0.007 | 0.001 | 2.20 | 0.06 | 0.23 | 0.060 | 0.00034 | 0.00107 | 0.00018 | 0.00105 | 0.00259 |
| LK38 | 2.5 | 0.13 | 0.003 | 0.001 | 1.62 | 0.04 | 0.18 | 0.045 | 0.00026 | 0.00009 | 0.00005 | 0.00053 | 0.00037 |
| LK39 | 1.9 | 0.09 | 0.002 | 0.017 | 1.26 | 0.09 | 0.19 | 0.005 | 0.00013 | 0.00072 | 0.00002 | 0.00211 | 0.00222 |
| LK40 | 2.0 | 0.10 | 0.002 | 0.001 | 1.24 | 0.03 | 0.14 | 0.033 | 0.00023 | 0.00018 | 0.00004 | 0.00026 | 0.00037 |
| LK41 | 1.9 | 0.09 | 0.003 | 0.007 | 1.20 | 0.05 | 0.14 | 0.035 | 0.00018 | 0.00018 | 0.00004 | 0.00105 | 0.00037 |
| LK42 | 1.9 | 0.11 | 0.003 | 0.001 | 1.26 | 0.03 | 0.14 | 0.033 | 0.00023 | 0.00018 | 0.00016 | 0.00053 | 0.00111 |
| LK43 | 2.1 | 0.09 | 0.004 | 0.001 | 1.34 | 0.04 | 0.15 | 0.040 | 0.00023 | 0.00036 | 0.00002 | 0.00158 | 0.00111 |
| LK44 | 2.8 | 0.12 | 0.003 | 0.003 | 1.81 | 0.05 | 0.21 | 0.045 | 0.00030 | 0.00197 | 0.00004 | 0.00053 | 0.00556 |

**Table S2. Field parameters, dissolved organic carbon concentration and isotope results. N/A represents data which has not been analysed and is not available.**

| Sample ID | Distance from  west coast (km) | Elevation (m) | | Temp (°C) | DO  (mg L^-1^) | Cond  (µS cm^-1^) | pH | Eh (mV) | DOC  (mg L^-1^) | 𝛿 ^13^C_DOC_ | 𝛿 ^13^C_DIC_ | 𝛿 ^18^O | 𝛿 ^2^H | ^87^Sr/^86^Sr |
| --- | --- | --- | --- | --- | --- | --- | --- | --- | --- | --- | --- | --- | --- | --- |
| LK1 | 2.48 | | 217 | 7.7 | 10.8 | 149.6 | 7.95 | 105.1 | 4.3 | -28.9 | -5 | -2.8 | -19.4 | N/A |
| LK2 | 1.31 | | 216 | 10 | 11.67 | 148.8 | 7.54 | 130 | 1.3 | -28.5 | -6.9 | -2.9 | -20.3 | 0.70816 |
| LK3 | 2.14 | | 185 | 11.4 | 9.5 | 291.5 | 6.51 | 292 | 5 | -25.5 | -22.1 | 0.7 | -1.9 | N/A |
| LK4 | 3.27 | | 314 | 7.8 | 11.33 | 128.4 | 7.94 | 132.7 | 1.7 | -27.2 | -2.4 | -3.1 | -22.9 | N/A |
| LK6 | 1.01 | | 169 | 11.2 | 11.15 | 209.3 | 7.34 | 243.3 | 4.1 | -26.5 | -11.9 | -1.5 | -12.5 | 0.70866 |
| LK7 | 2.86 | | 256 | 8.6 | 10.34 | 139.2 | 8 | 145.6 | 1.8 | -27.1 | -4.7 | -3.9 | -26.3 | N/A |
| LK8 | 2.03 | | 188 |  | 10.44 | 113.1 | 8.5 | 254.7 | 2 | -27.5 | -18.8 | -1.6 | -13.1 | N/A |
| LK9 | 2.79 | | 138 | 8.9 | 11.61 | 202.7 | 9.21 | 125.8 | 2.5 | -25.6 | -9.3 | -3.7 | -24.6 | N/A |
| LK10 | 1.49 | | 198 | 10 | 11.99 | 210.8 | 6.93 | 234.6 | 2.5 | -26 | -13.4 | -1.7 | -13.5 | N/A |
| LK11 | 2.87 | | 173 | 10.3 | 10.68 | 137.2 | 6.64 | 214.6 | 1.4 | -28.3 | -13.9 | -2.6 | -18 | N/A |
| LK12 | 3.25 | | 163 | 9.9 | 10.81 | 122 | 6.41 | 225 | 1.1 | -28.8 | -16.7 | -1.5 | -12.9 | N/A |
| LK14 | 3.51 | | 163 | 8.8 | 11.49 | 121 | 6.94 | 164.8 | 0.7 | -30.8 | -13.9 | -3.2 | -21.7 | 0.70766 |
| LK15 | 4 | | 163 | 8 | 11.31 | 122.5 | 6.75 | 254.6 | 2.2 | -26.6 | -18.8 | -2.1 | -15.6 | N/A |
| LK16 | 3.66 | | 95 | 9.4 | 10.44 | 143 | 7.4 | 137.1 | 3.4 | -30.3 | -16 | -5.3 | -34.9 | N/A |
| LK18 | 1 | | 214 | 15.8 | 8.56 | 195.9 | 6.33 | 184.5 | 2.2 | -28.5 | -21.7 | -2.4 | -18.7 | N/A |
| LK19 | 2.21 | | 230 | 10.4 | 10.64 | 139.8 | 7.66 | 127.2 | 2.7 | -36.7 | -6 | -2.7 | -18.6 | N/A |
| LK20 | 3.82 | | 126 | 9.7 | 10.37 | 122.2 | 7.4 | 154.4 | 3 | -28.8 | -11.9 | -2.4 | -17.7 | N/A |
| LK21 | 1.9 | | 251 | 9.5 | 10.85 | 129.8 | 6.8 | 174 | 1.5 | -28.4 | -15.4 | -2.5 | -17.1 | N/A |
| LK22 | 4.14 | | 103 | 10.5 | 10.5 | 115.7 | 7.04 | 123.7 | 6.2 | -29.9 | -12.4 | -2.3 | -17.6 | N/A |
| LK23 | 3.32 | | 201 | 10 | 11.22 | 152.7 | 7.98 | 116.2 | 1.4 | -30.6 | -3.6 | -4.8 | -31.3 | N/A |
| LK24 | 1.51 | | 144 | 10.7 | 11.64 | 174 | 7.83 | 193.1 | 3.5 | -28 | -6.9 | -3.5 | -24.1 | N/A |
| LK25 | 1.42 | | 141 | 12.7 | 9.84 | 100.9 | 7.89 | 171.5 | 2.2 | -27.8 | -12.3 | -4.2 | -27.4 | N/A |
| LK26 | 4.69 | | 9 | 9.6 | 10.03 | 156.1 | 7.57 | 115.6 | 3.2 | -28.8 | -15.1 | -5 | -32.9 | N/A |
| LK27 | 2.01 | | 189 | 8.4 | 11.07 | 157.2 | 7.5 | 146.5 | 2.4 | -35.7 | -2.6 | -3.8 | -26.1 | N/A |
| LK28 | 1.88 | | 223 | 7.2 | 11.8 | 100.8 | 6.44 | 166.8 | 0.8 | -27.7 | -15.9 | -1.9 | -13.7 | N/A |
| LK29 | 1.61 | | 206 | 7.6 | 11.53 | 135.4 | 7.59 | 162.8 | 0.9 | -27 | -1.5 | -3.4 | -22.5 | 0.70694 |
| LK30 | 2.02 | | 176 | 7.5 | 11.61 | 127.1 | 7.63 | 161.1 | 3.1 | -27.9 | -8.3 | -4.4 | -28.8 | N/A |
| LK31 | 1.24 | | 260 | 9.6 | 10.45 | 154.9 | 7.53 | 127 | 1.4 | -27 | -11.2 | -2.9 | -20.1 | 0.70831 |
| LK32 | 3.05 | | 191 | 7.6 | 11.48 | 102.9 | 6.94 | 163.3 | 1.1 | -29.1 | -13.9 | -2.9 | -19.8 | N/A |
| LK34 | 2.8 | | 126 | 7.9 | 11.7 | 124.5 | 7.99 | 150 | 2.6 | -24.8 | -8.3 | -2.6 | -19.1 | 0.70785 |
| LK35 | 1.77 | | 223 | 9.5 | 11.71 | 189.6 | 8.65 | 113.7 | 2.1 | -23.7 | -8.3 | -2.7 | -20 | N/A |
| LK36 | 1.27 | | 189 | 10.3 | 10.67 | 200.3 | 7.56 | 137.6 | 1.4 | -30 | -7.5 | -3.3 | -22.2 | N/A |
| LK37 | 1.08 | | 196 | 10.5 | 10.6 | 244.5 | 6.44 | 173.4 | 3.2 | -27.3 | -23.1 | -1.2 | -10.9 | 0.70894 |
| LK38 | 1.27 | | 185 | 10.3 | 11.24 | 180.4 | 6.49 | 174.8 | 1.4 | -29 | -16.7 | -2.4 | -17.9 | 0.70908 |
| LK39 | 2.58 | | 184 | 9.5 | 12.64 | 142.6 | 8.98 | 163 | 3.2 | -25.7 | -8.7 | -3.4 | -23.8 | N/A |
| LK40 | 1.63 | | 208 | 8.8 | 10.54 | 136.7 | 6.61 | 177.4 | 1.4 | -27.5 | -11.2 | -2.7 | -20.3 | N/A |
| LK41 | 1.76 | | 306 | 10.3 | 10.75 | 139.1 | 7.15 | 169.2 | 1.2 | -28.5 | -7.2 | -3.6 | -24.7 | N/A |
| LK42 | 1.57 | | 278 | 6.8 | 11.61 | 123.8 | 5.99 | 212.1 | 1.3 | -28.9 | -18.8 | -2.2 | -16.1 | N/A |
| LK43 | 2.27 | | 252 | 6.9 | 11.21 | 135.5 | 6.73 | 207.2 | 1.8 | -25.2 | -16.2 | -2.8 | -20.2 | 0.70877 |
| LK44 | 1 | | 200 | 9.3 | 10.16 | 191 | 7.15 | 156.5 | 6.8 | -28.9 | -19 | -1.9 | -15.3 | 0.70904 |

**Table S3. Spearman correlations (⍴) and significance values (*p*-values) for variables. P-values shown in bold represent significant relationships at α = 0.95.**

| Variables | ⍴ | *p*-value |
| --- | --- | --- |
| Cl, Na | 0.95 | **< 2.2 x 10^-16^** |
| Cl, SO_4_ | 0.80 | **4.0 x 10^-10^** |
| Cl, Mg | 0.75 | **2.4 x 10^-8^** |
| Na, SO_4_ | 0.78 | **3.2 x 10^-9^** |
| Na, Mg | 0.72 | **1.9 x 10^-7^** |
| SO_4_, Mg | 0.63 | **1.4 x 10^-5^** |
| Cl, Br | 0.76 | **1.8 x 10^-8^** |
| Cl, K | 0.68 | **1.7 x 10^-6^** |
| Cl, Sr | 0.71 | **2.2 x 10^-7^** |
| Na, Br | 0.68 | **1.1 x 10^-6^** |
| Na, K | 0.58 | **7.5 x 10^-5^** |
| Na, Sr | 0.67 | **2.6 x 10^-6^** |
| SO_4_, Br | 0.73 | **1.0 x 10^-7^** |
| SO_4_, K | 0.67 | **2.8 x 10^-6^** |
| SO_4_, Sr | 0.69 | **7.4 x 10^-7^** |
| Br, Mg | 0.44 | **4.3 x 10^-3^** |
| Br, K | 0.79 | **9.9 x 10^-10^** |
| Br, Sr | 0.63 | **1.5 x 10^-5^** |
| Mg, K | 0.46 | **3.1 x 10^-3^** |
| Mg, Sr | 0.59 | **7.2 x 10^-5^** |
| K, SiO_2_ | -0.44 | **4.4 x 10^-3^** |
| Ca, F | 0.72 | **1.9 x 10^-7^** |
| Ca, SiO_2_ | 0.78 | **2.4 x 10^-9^** |
| F, SiO_2_ | 0.49 | **1.3 x 10^-3^** |
| K, Ca | -0.35 | **2.5 x 10^-2^** |
| K, F | -0.26 | 0.1 |
| Cl, SiO_2_ | -0.14 | 0.4 |
| Cl, F | 0.24 | 0.1 |

**Table S4. Variables significantly influencing PCA cluster groups. Mean and standard deviation (σ) values have been centred and scaled to the standard deviation for each variable.**

|  | v-test | Mean in category | Overall mean | σ in category | Overall σ | p-value |
| --- | --- | --- | --- | --- | --- | --- |
| Cluster Group 1 | | | | | | |
| SO_4_ | 5.15 | 0.1514 | 0.1035 | 0.0275 | 0.0290 | <0.01 |
| Cl | 5.12 | 2.8580 | 1.9997 | 0.4601 | 0.5233 | <0.01 |
| Na | 5.11 | 1.9079 | 1.3504 | 0.3075 | 0.3409 | <0.01 |
| EC | 4.86 | 215.46 | 152.81 | 33.77 | 40.2554 | <0.01 |
| Br | 4.73 | 0.0040 | 0.0025 | 0.0011 | 0.0010 | <0.01 |
| Sr | 4.58 | 0.0003 | 0.0002 | 0.0001 | 0.0001 | <0.01 |
| K | 4.40 | 0.0504 | 0.0319 | 0.0072 | 0.0131 | <0.01 |
| Mg | 3.46 | 0.2096 | 0.1627 | 0.0419 | 0.0423 | <0.01 |
| ^18^O | 3.14 | -1.7125 | -2.8275 | 1.0971 | 1.1093 | <0.01 |
| ^2^H | 3.00 | -14.1125 | -20.1125 | 5.7516 | 6.2407 | <0.01 |
| Al | 2.41 | 0.0025 | 0.0015 | 0.0020 | 0.0013 | 0.02 |
| DOC concentration | 2.10 | 3.3250 | 2.4000 | 1.7626 | 1.3740 | 0.04 |
| pH | -2.14 | 6.8438 | 7.3483 | 0.4365 | 0.7361 | 0.03 |
| ^13^C_DIC_ | -2.74 | -16.9250 | -11.9375 | 5.2270 | 5.6780 | <0.01 |
| Distance from west coast | -3.23 | 1.2825 | 2.2873 | 0.3629 | 0.9713 | <0.01 |
| d-excess | -3.33 | -0.4125 | 2.5075 | 3.0909 | 2.7399 | <0.01 |
| Cluster Group 2 | | | | | | |
| Ca | 5.24 | 0.1927 | 0.0854 | 0.0547 | 0.0639 | <0.01 |
| F | 5.12 | 0.0034 | 0.0014 | 0.0013 | 0.0012 | <0.01 |
| SiO_2_ | 4.60 | 0.0414 | 0.0126 | 0.0241 | 0.0195 | <0.01 |
| d-excess | 3.78 | 5.8250 | 2.5075 | 1.4489 | 2.7399 | <0.01 |
| pH | 3.02 | 8.0613 | 7.3483 | 0.6243 | 0.7361 | <0.01 |
| Fe | 3.00 | 0.0015 | 0.0007 | 0.0011 | 0.0008 | <0.01 |
| Sr | -2.48 | 0.0002 | 0.0002 | 0.0000 | 0.0001 | 0.01 |
| Elevation | -3.05 | 136.0000 | 191.2250 | 57.0438 | 56.5621 | <0.01 |
| ^18^O | -4.11 | -4.2875 | -2.8275 | 0.6679 | 1.1093 | <0.01 |
| K | -4.13 | 0.0145 | 0.0319 | 0.0082 | 0.0131 | <0.01 |
| ^2^H | -4.18 | -28.4750 | -20.1125 | 3.9704 | 6.2407 | <0.01 |
| Cluster Group 3 | | | | | | |
| Elevation | 2.45 | 209.3750 | 191.2250 | 53.3829 | 56.5621 | 0.01 |
| Al | -1.99 | 0.0012 | 0.0015 | 0.0008 | 0.0013 | 0.05 |
| SiO_2_ | -2.42 | 0.0065 | 0.0126 | 0.0095 | 0.0195 | 0.02 |
| Br | -2.42 | 0.0022 | 0.0025 | 0.0005 | 0.0010 | 0.02 |
| DOC concentration | -2.48 | 1.9542 | 2.4000 | 1.1906 | 1.3740 | 0.01 |
| Mg | -2.74 | 0.1476 | 0.1627 | 0.0272 | 0.0423 | 0.01 |
| Fe | -2.83 | 0.0004 | 0.0007 | 0.0004 | 0.0008 | <0.01 |
| SO_4_ | -2.87 | 0.0926 | 0.1035 | 0.0113 | 0.0290 | <0.01 |
| Ca | -3.25 | 0.0583 | 0.0854 | 0.0302 | 0.0639 | <0.01 |
| Cl | -3.46 | 1.7631 | 1.9997 | 0.2489 | 0.5233 | <0.01 |
| F | -3.48 | 0.0008 | 0.0014 | 0.0005 | 0.0012 | <0.01 |
| Conductivity | -3.78 | 132.9042 | 152.8125 | 18.5712 | 40.2554 | <0.01 |
| Na | -3.82 | 1.1802 | 1.3504 | 0.1478 | 0.3409 | <0.01 |


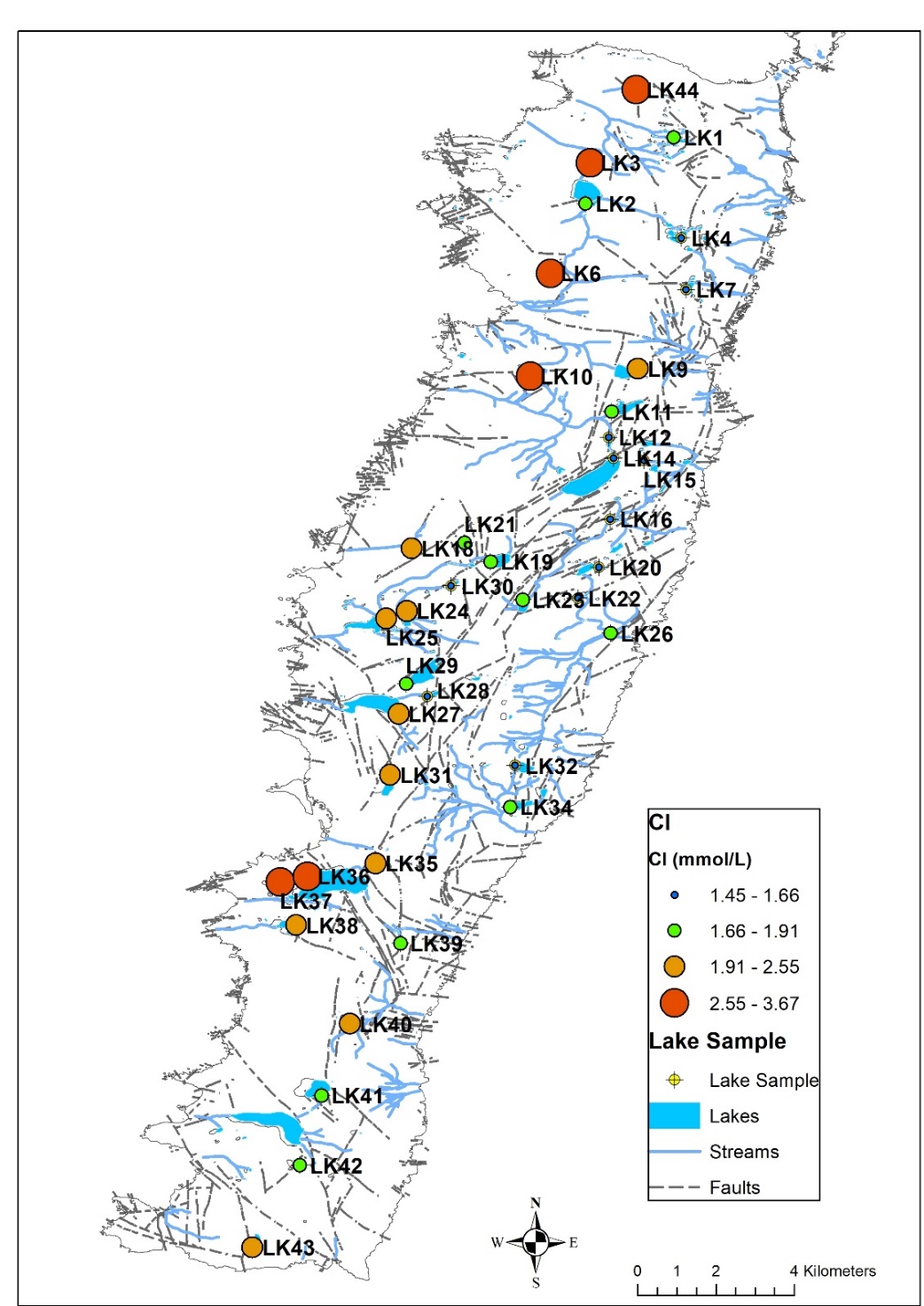

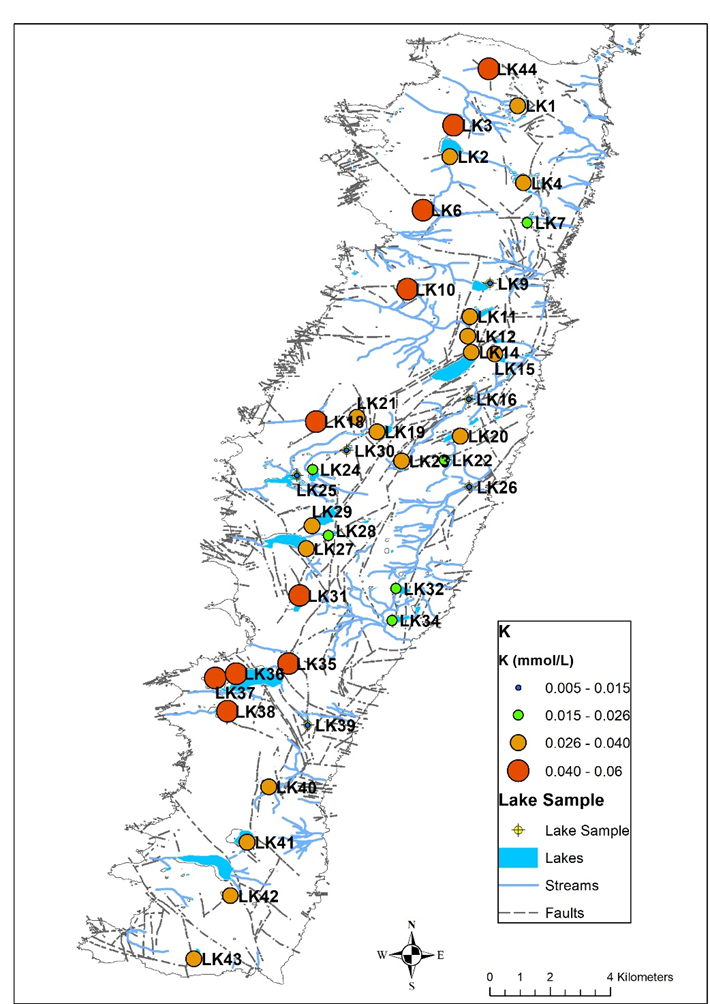


**Fig. S1 Chloride (Cl) andpotassium (K) concentrations at each sampling site. Samples where data is not available are shown as a yellow bullseye symbol. Coloured points represent lake sample locations, with point colour and size representing values shown in the legend.**


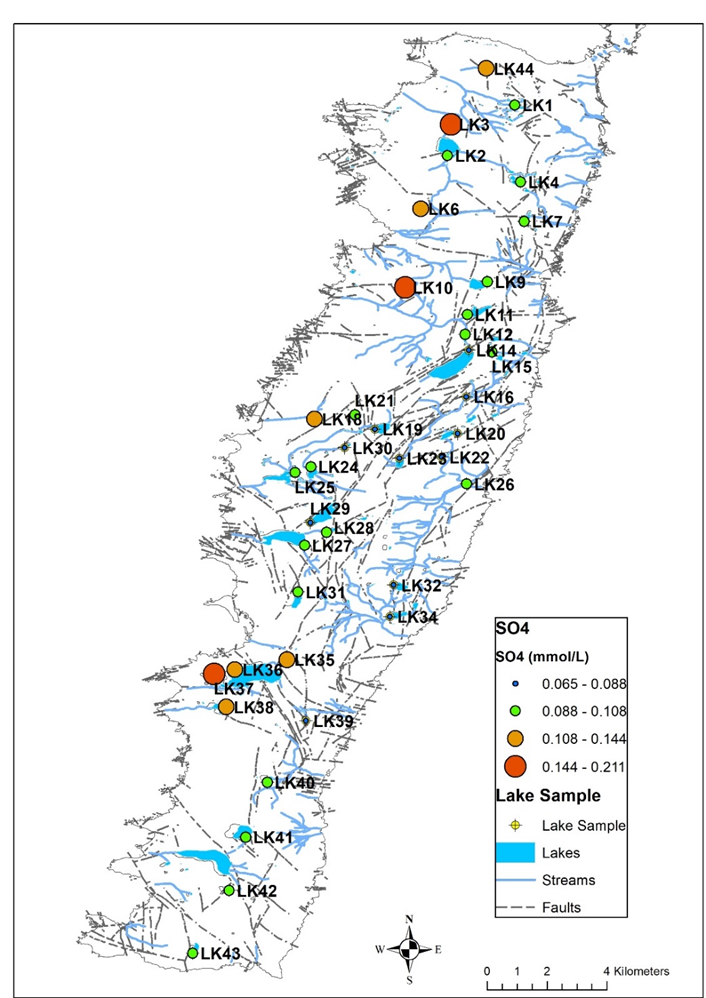

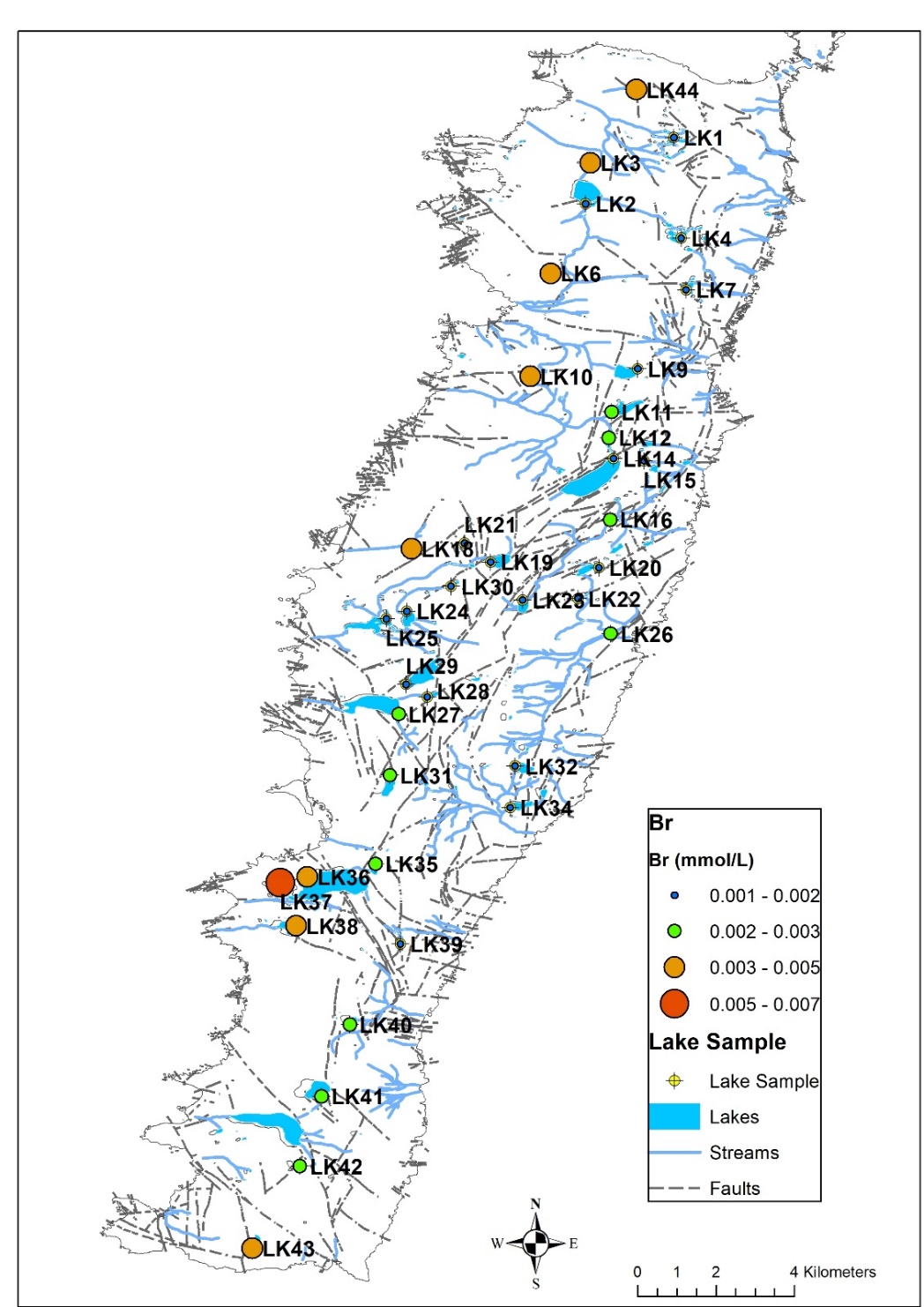


**Fig. S2 Sulphate (SO_4_) and bromium (Br) concentrations at each sampling site. Samples where data is not available are shown as a yellow bullseye symbol. Coloured points represent lake sample locations, with point colour and size representing values shown in the legend.**


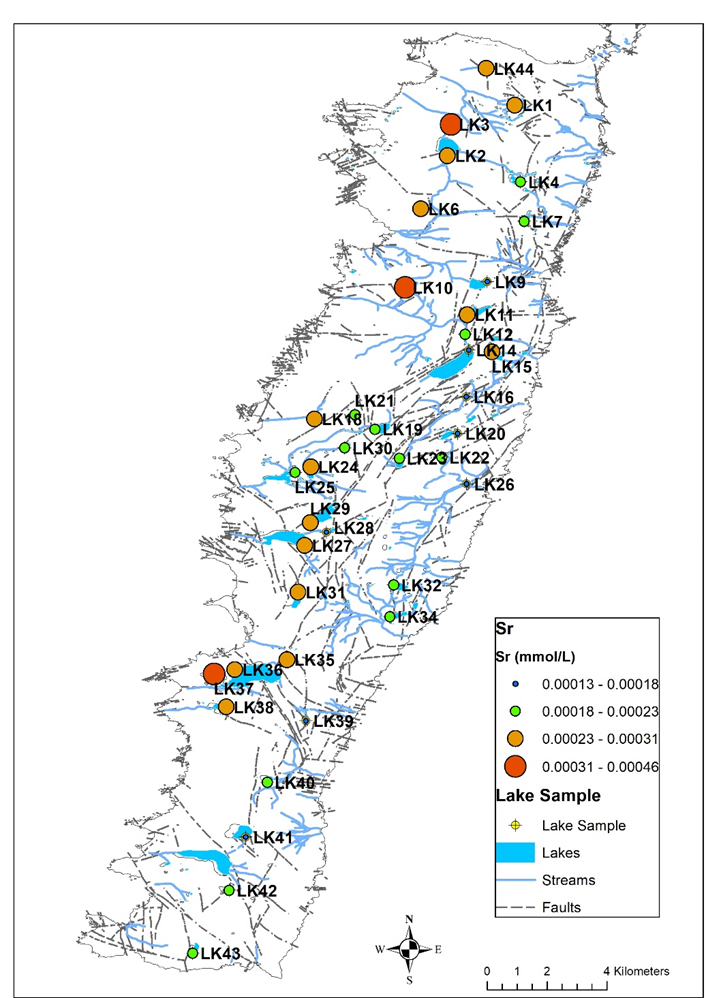

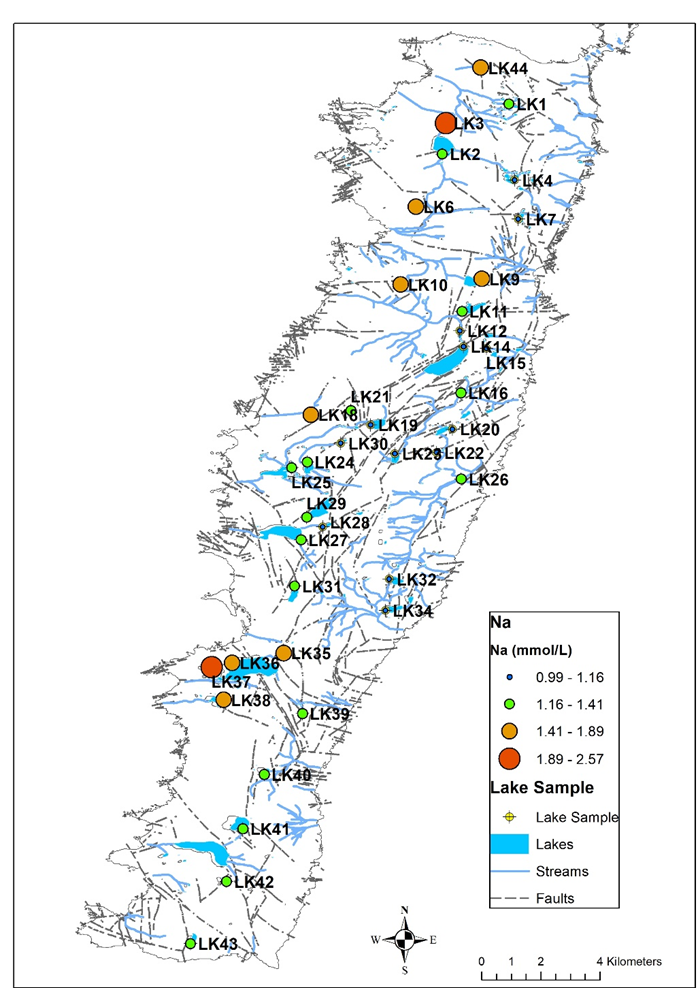


**Fig. S3 Strontium (Sr) and sodium (Na) concentrations at each sampling site. Samples where data is not available are shown as a yellow bullseye symbol. Coloured points represent lake sample locations, with point colour and size representing values shown in the legend.**


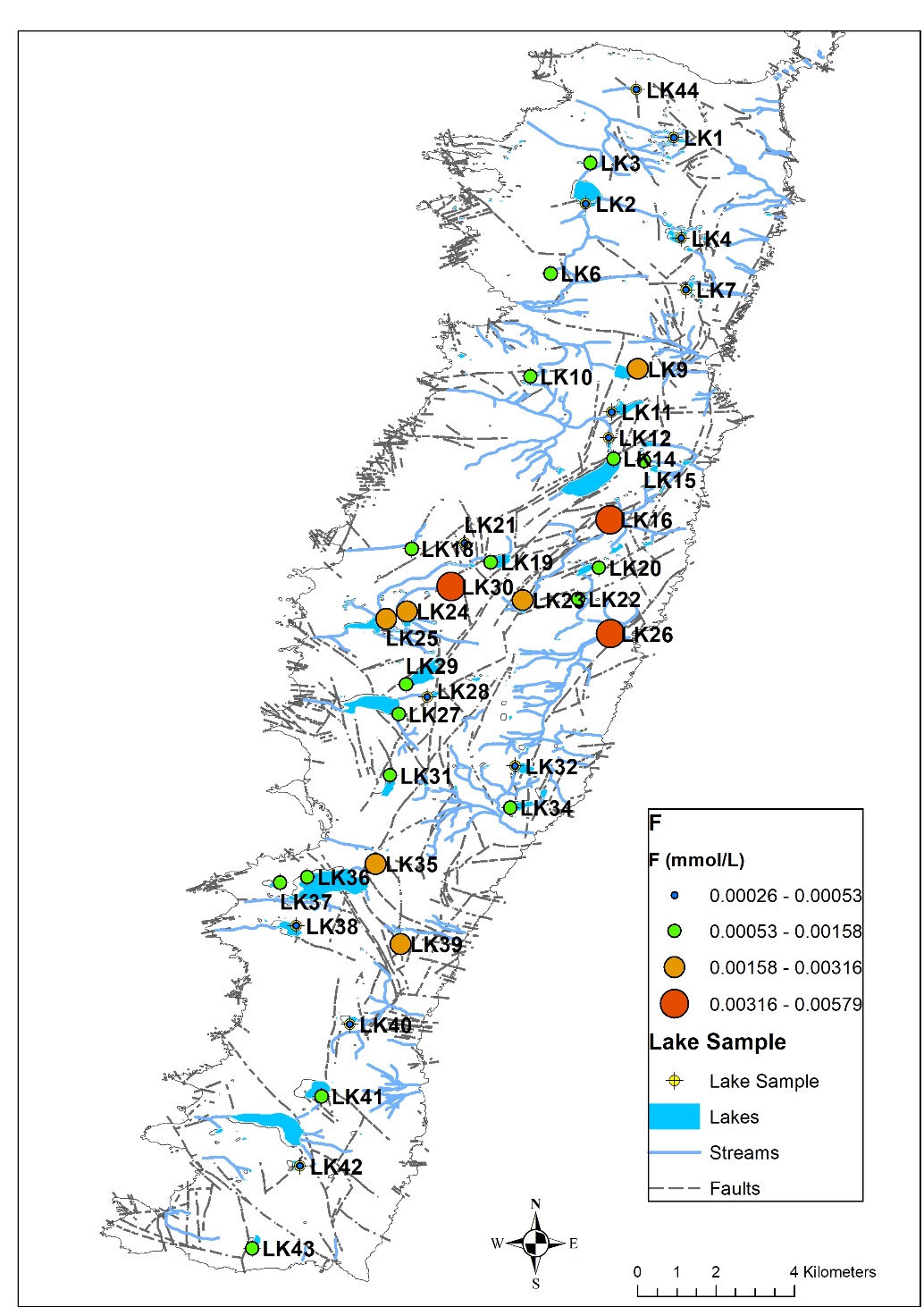

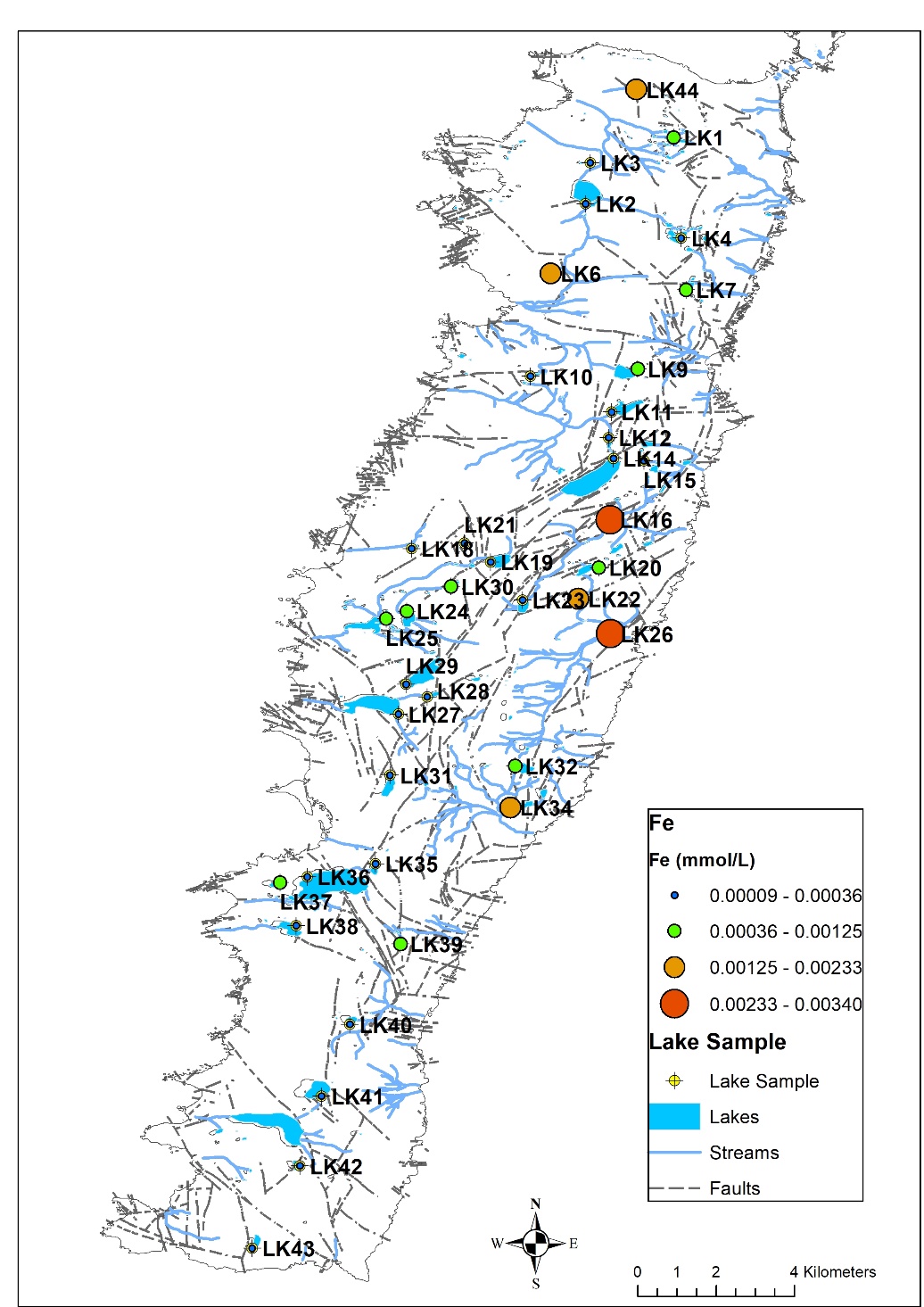


**Fig. S4 Fluoride (F) and iron (Fe) concentrations at each sampling site. Samples where data is not available are shown as a yellow bullseye symbol. Coloured points represent lake sample locations, with point colour and size representing values shown in the legend.**


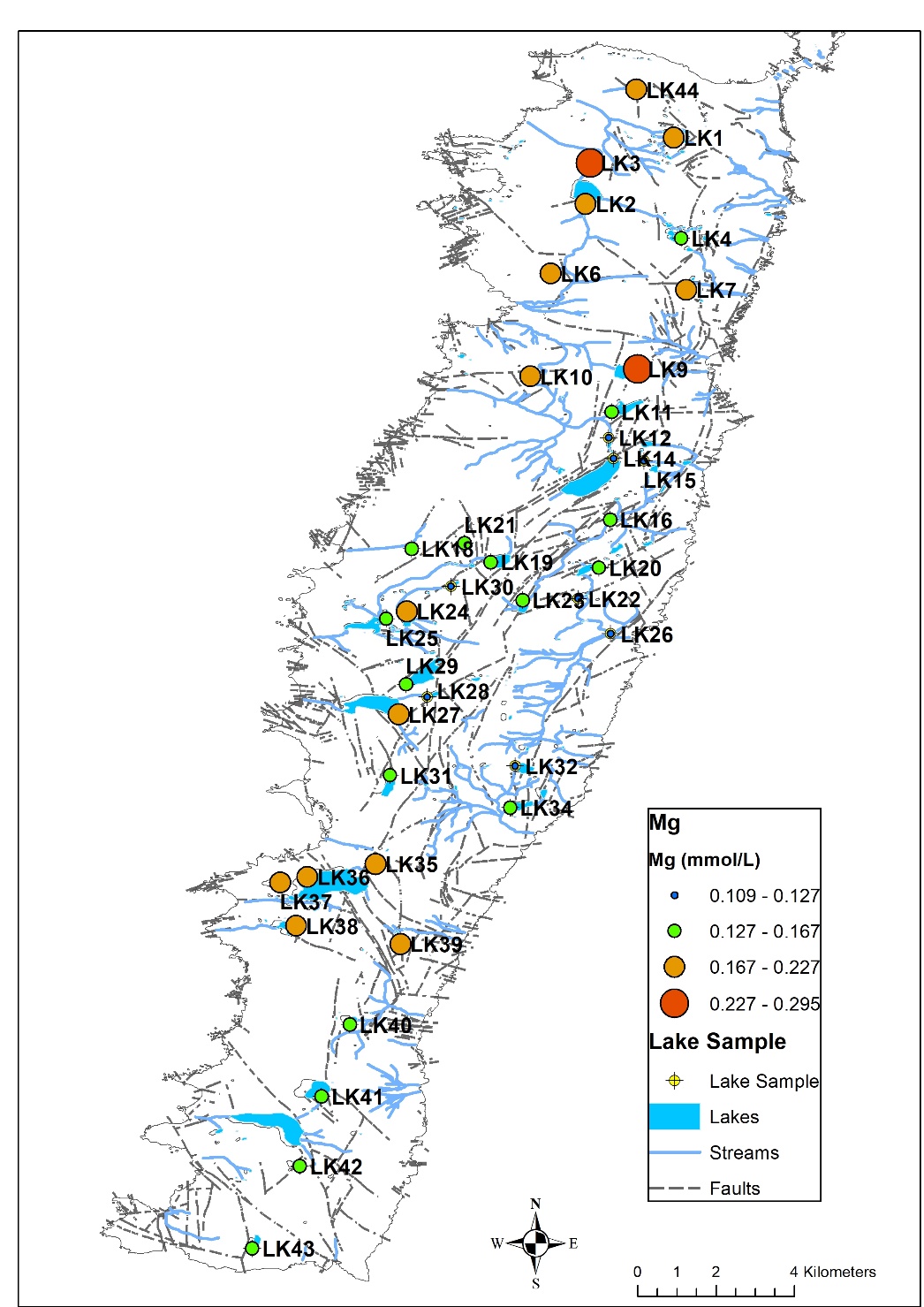

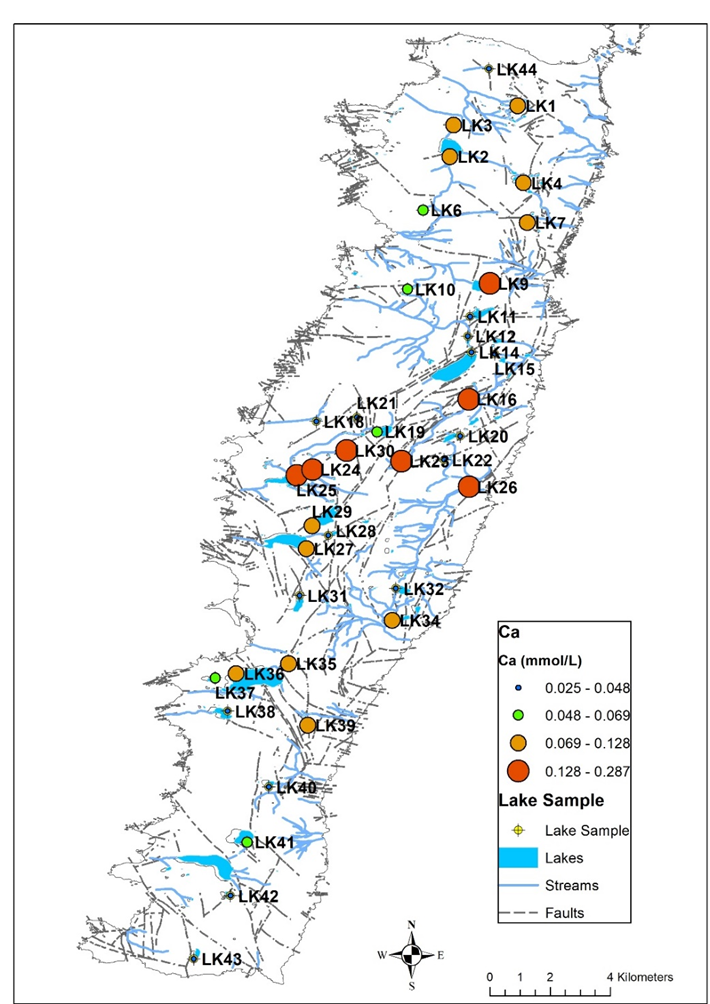


**Fig. S5. Magnesium (Mg) and Calcium (Ca) concentrations at each sampling site. Samples where data is not available are shown as a yellow bullseye symbol. Coloured points represent lake sample locations, with point colour and size representing values shown in the legend.**


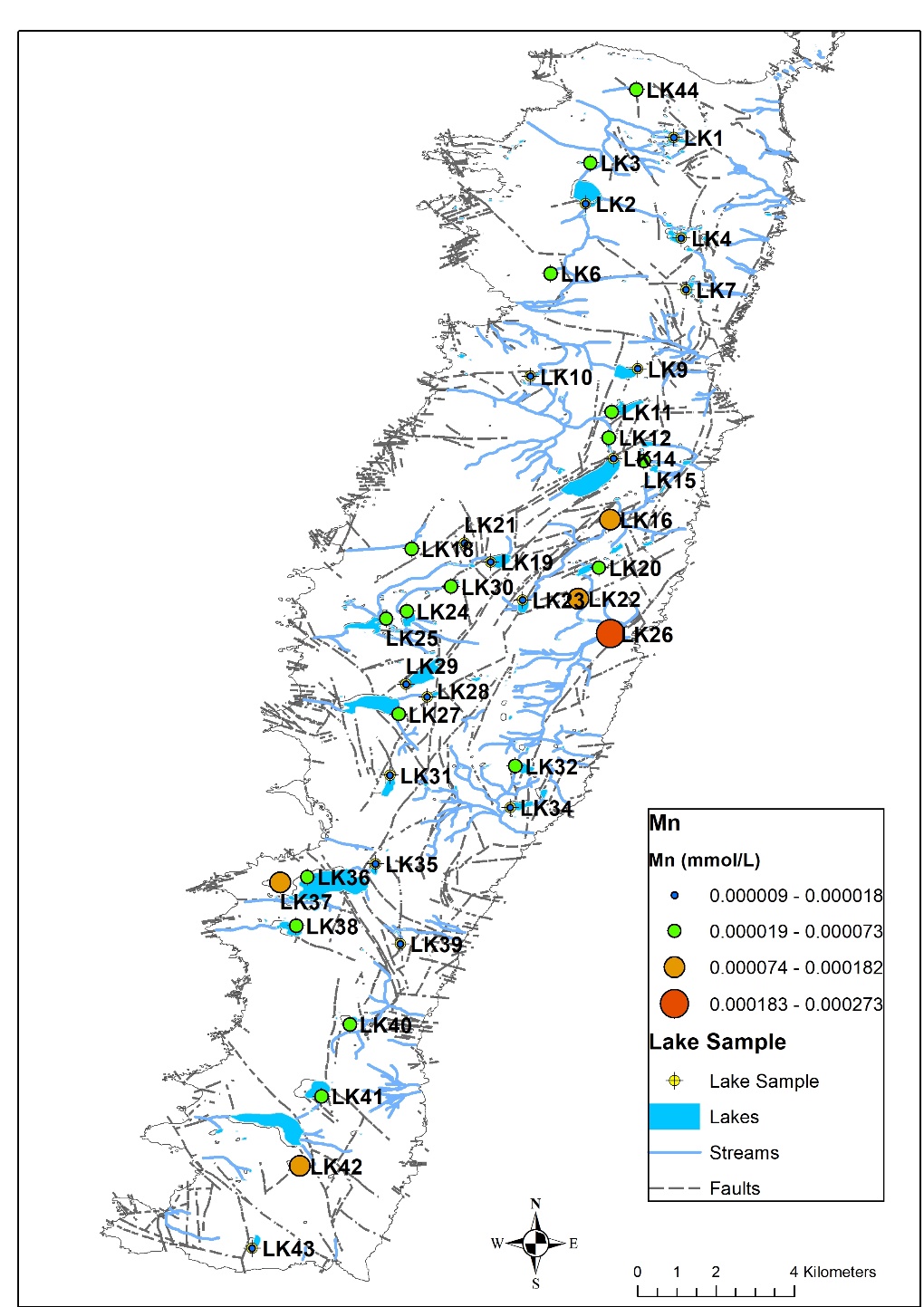

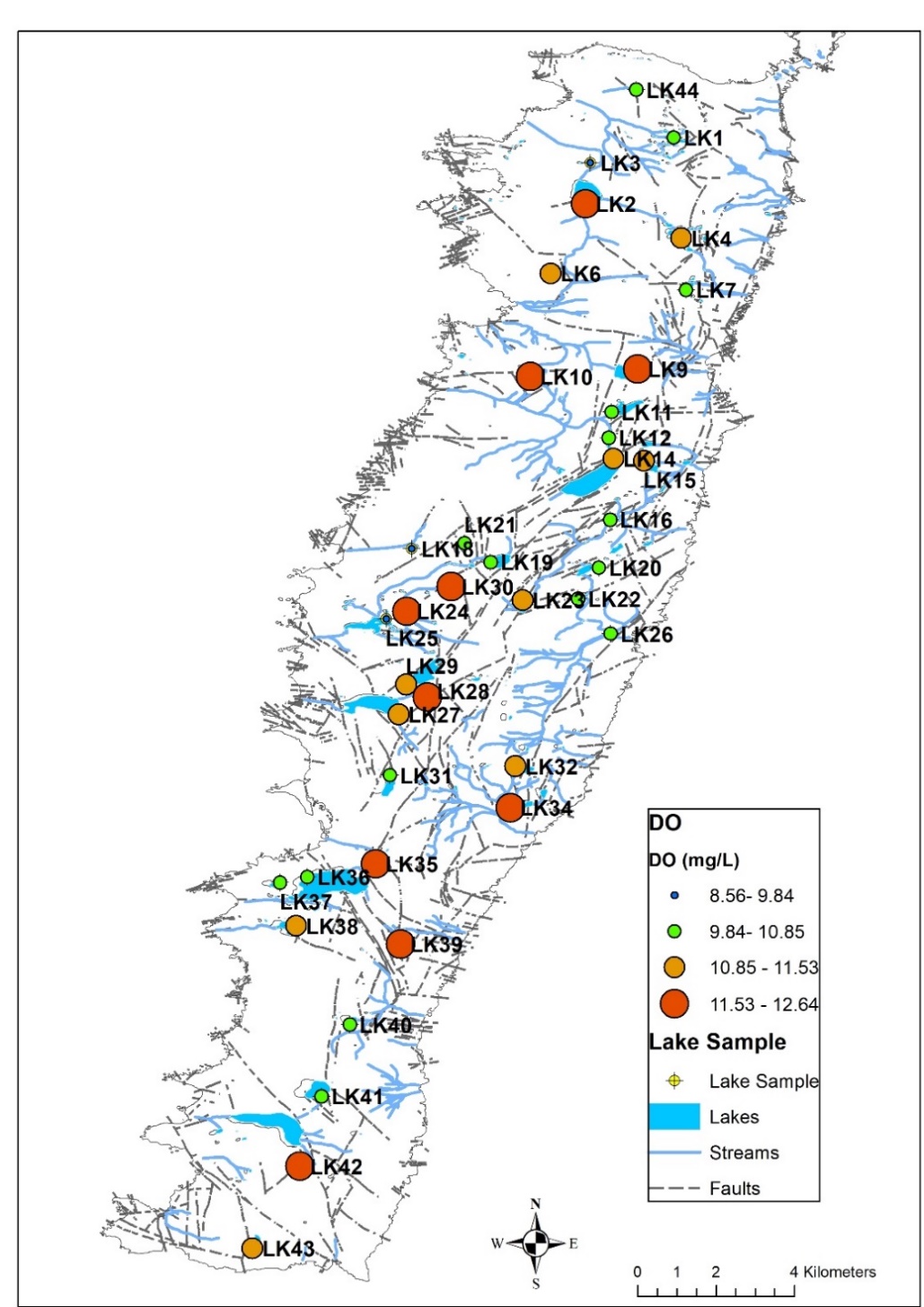


**Fig. S6 Manganese (Mn) and dissolved oxygen (DO) concentrations at each sampling site. Samples where data is not available are shown as a yellow bullseye symbol. Coloured points represent lake sample locations, with point colour and size representing values shown in the legend.**


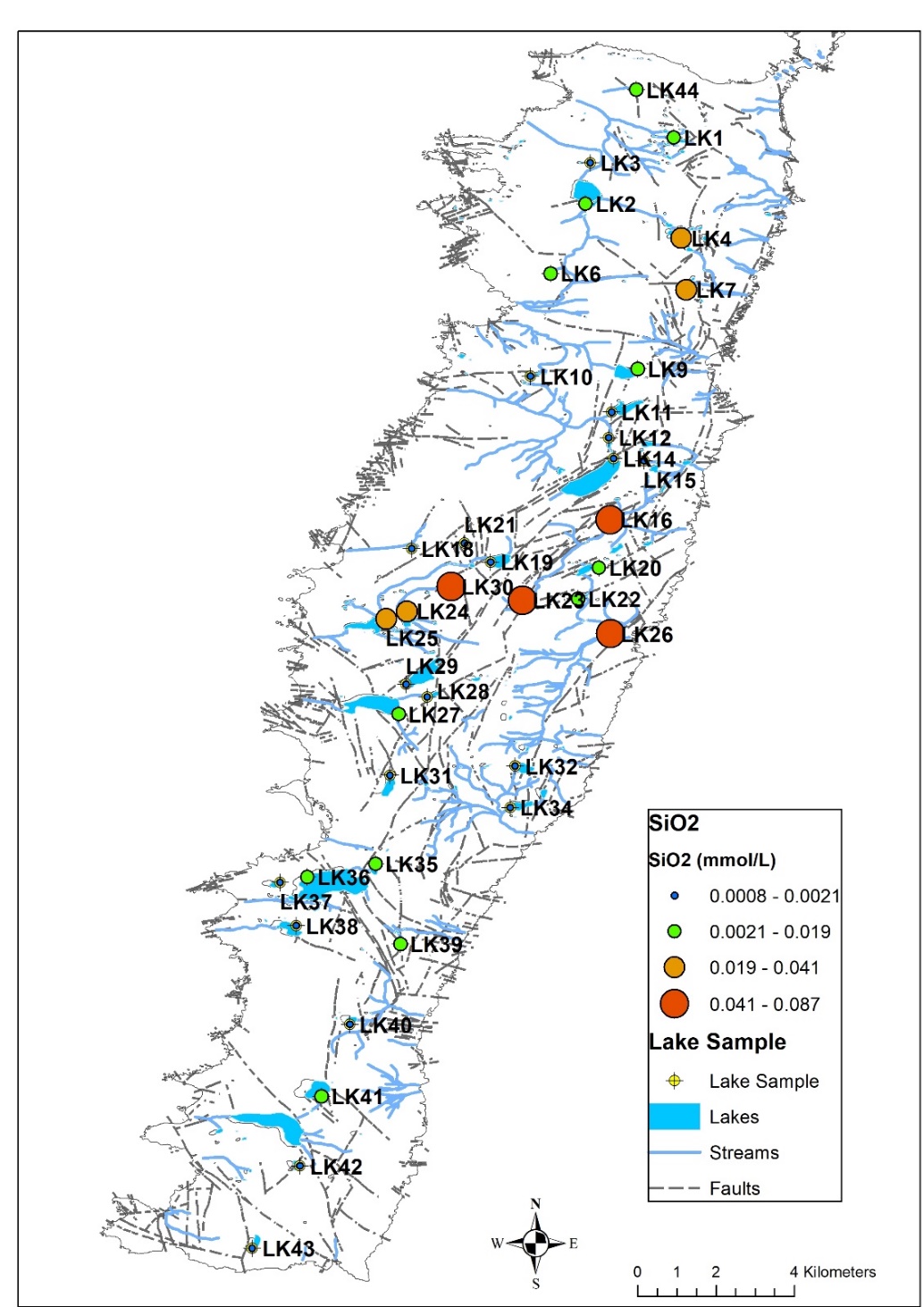

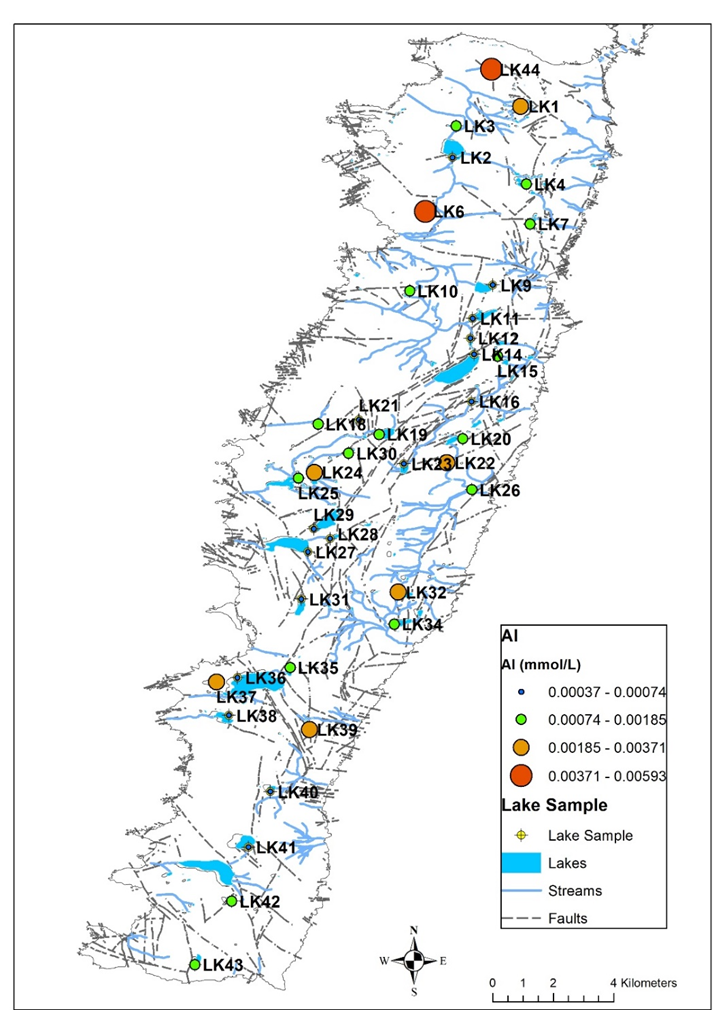


**Fig. S7 Silicon dioxide (SiO_2_) and aluminium (Al) concentrations at each sampling site. Samples where data is not available are shown as a yellow bullseye symbol. Coloured points represent lake sample locations, with point colour and size representing values shown in the legend.**


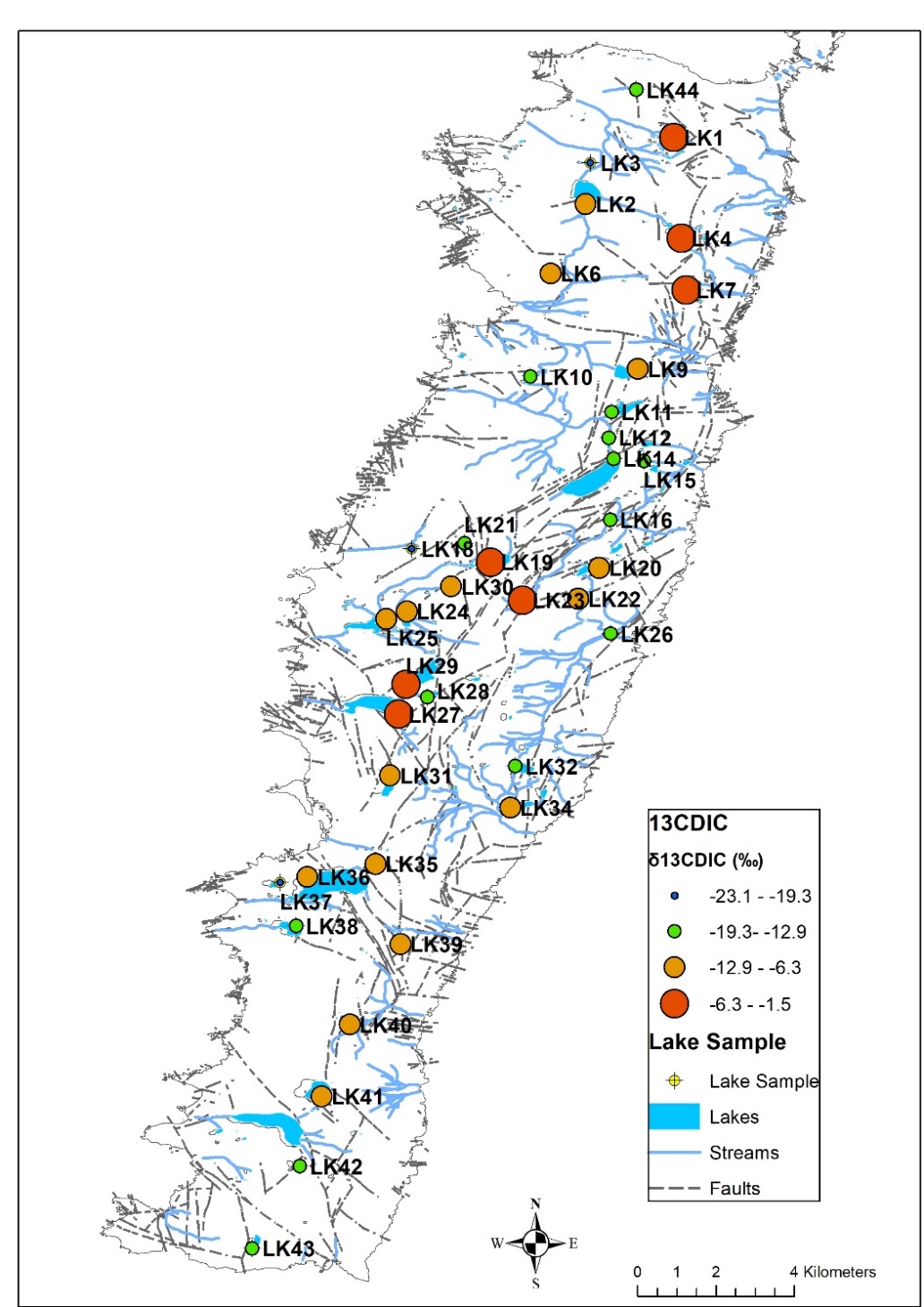

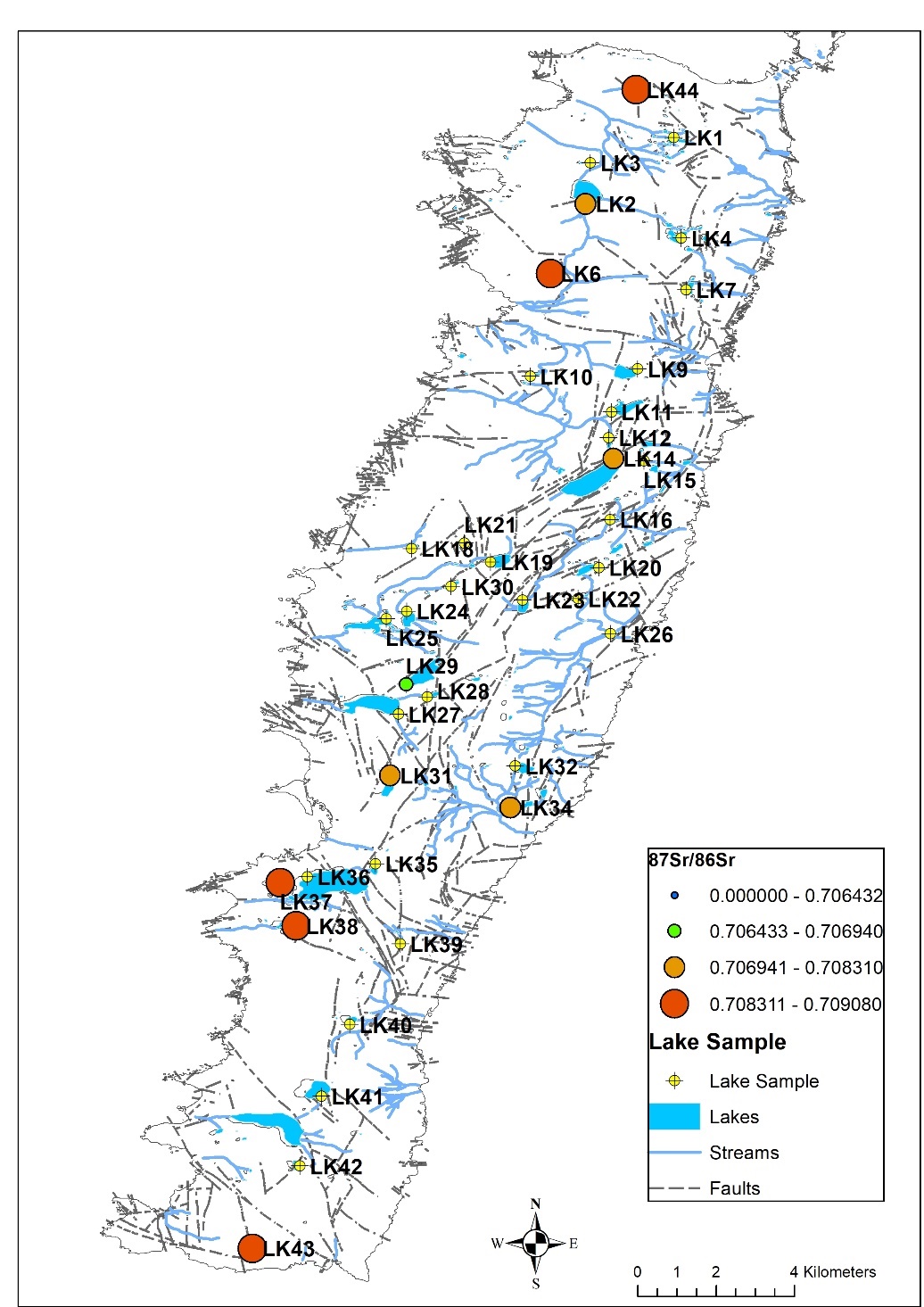


**Fig. S8 δ^13^C_DiC_ and ^87^Sr/^86^Sr ratios at each sampling site. Samples where data is not available are shown as a yellow bullseye symbol. Coloured points represent lake sample locations, with point colour and size representing values shown in the legend.**


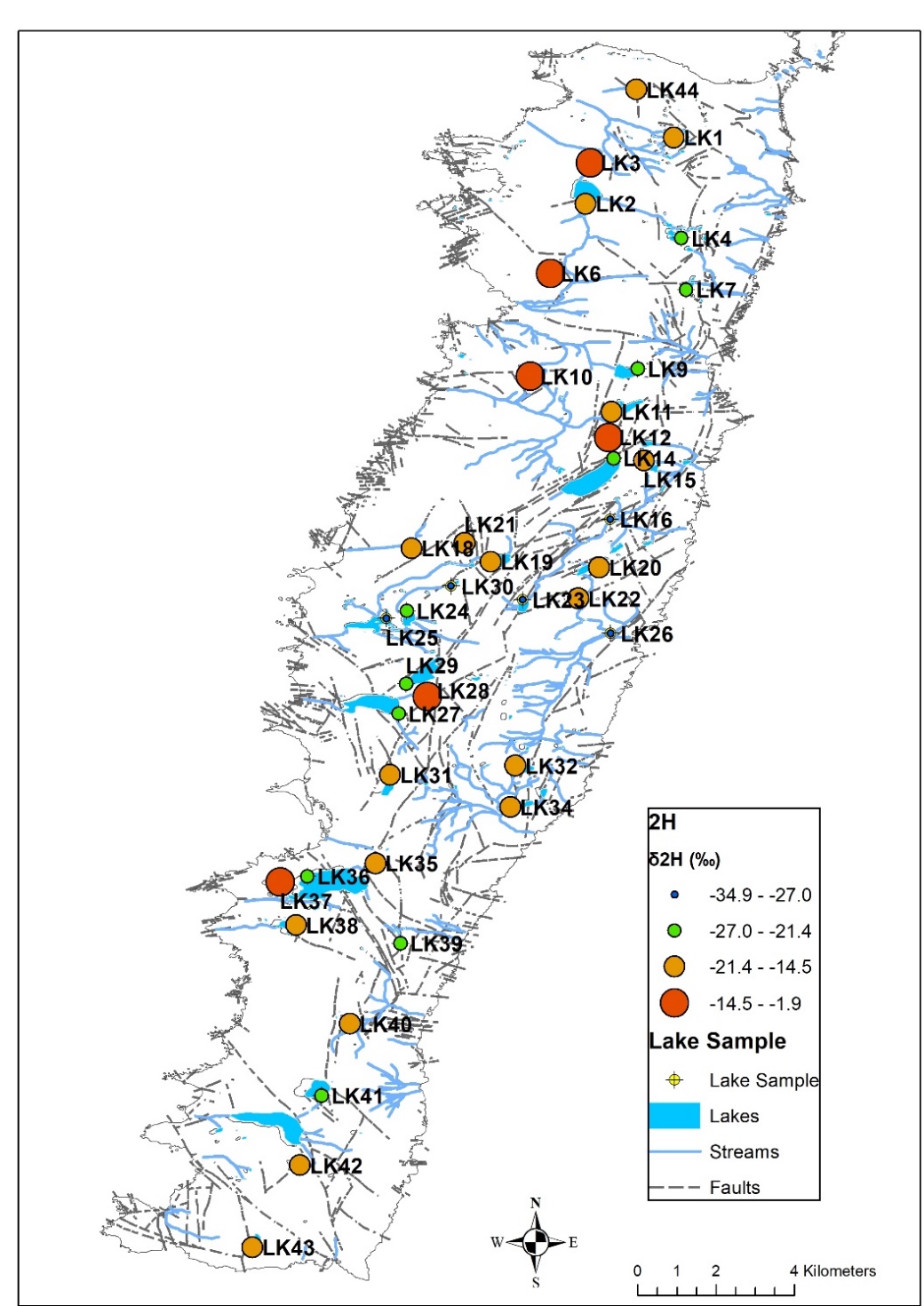

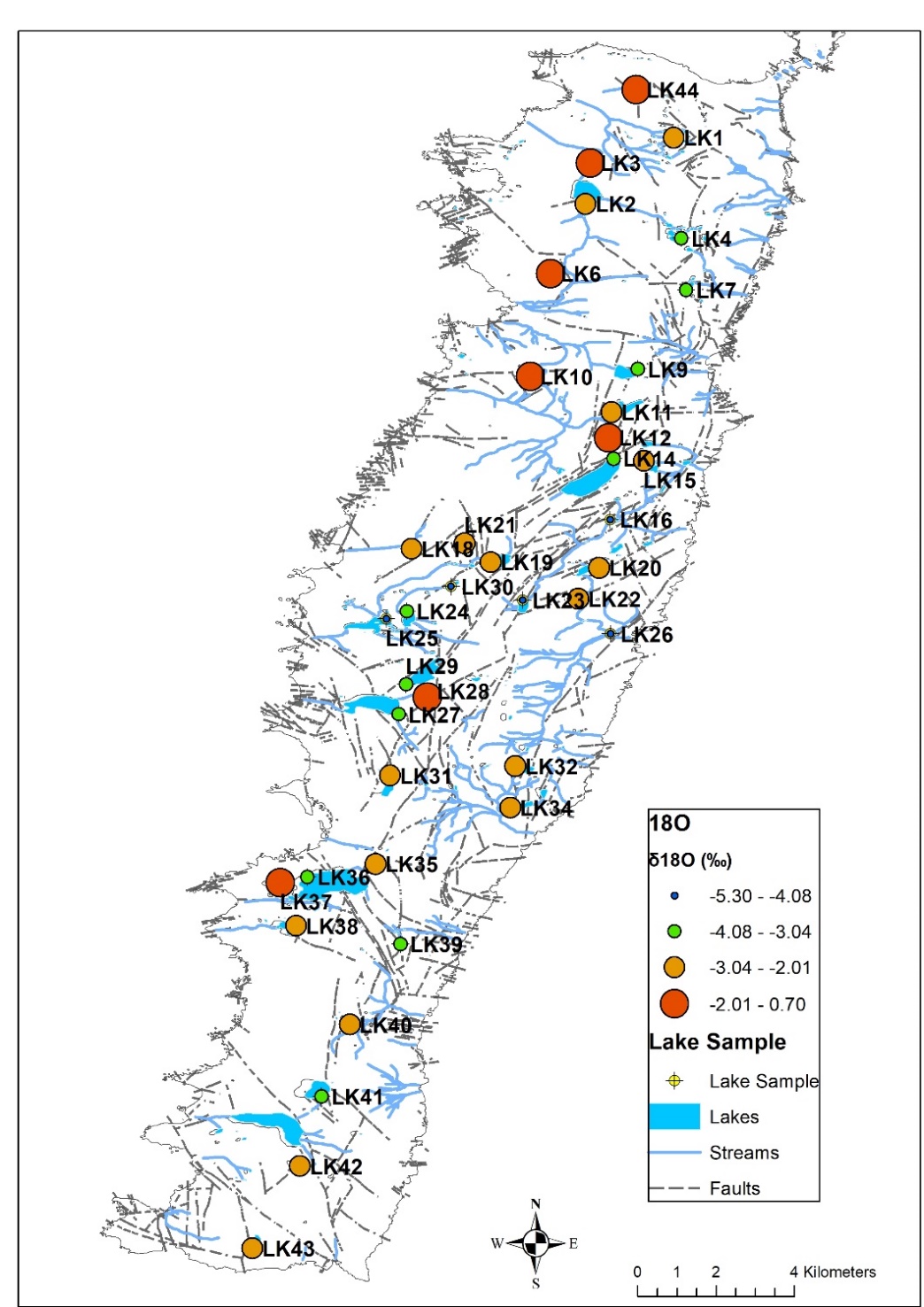


**Fig. S9 δ^2^H and δ^18^O at each sampling site. Samples where data is not available are shown as a yellow bullseye symbol. Coloured points represent lake sample locations, with point colour and size representing values shown in the legend.**


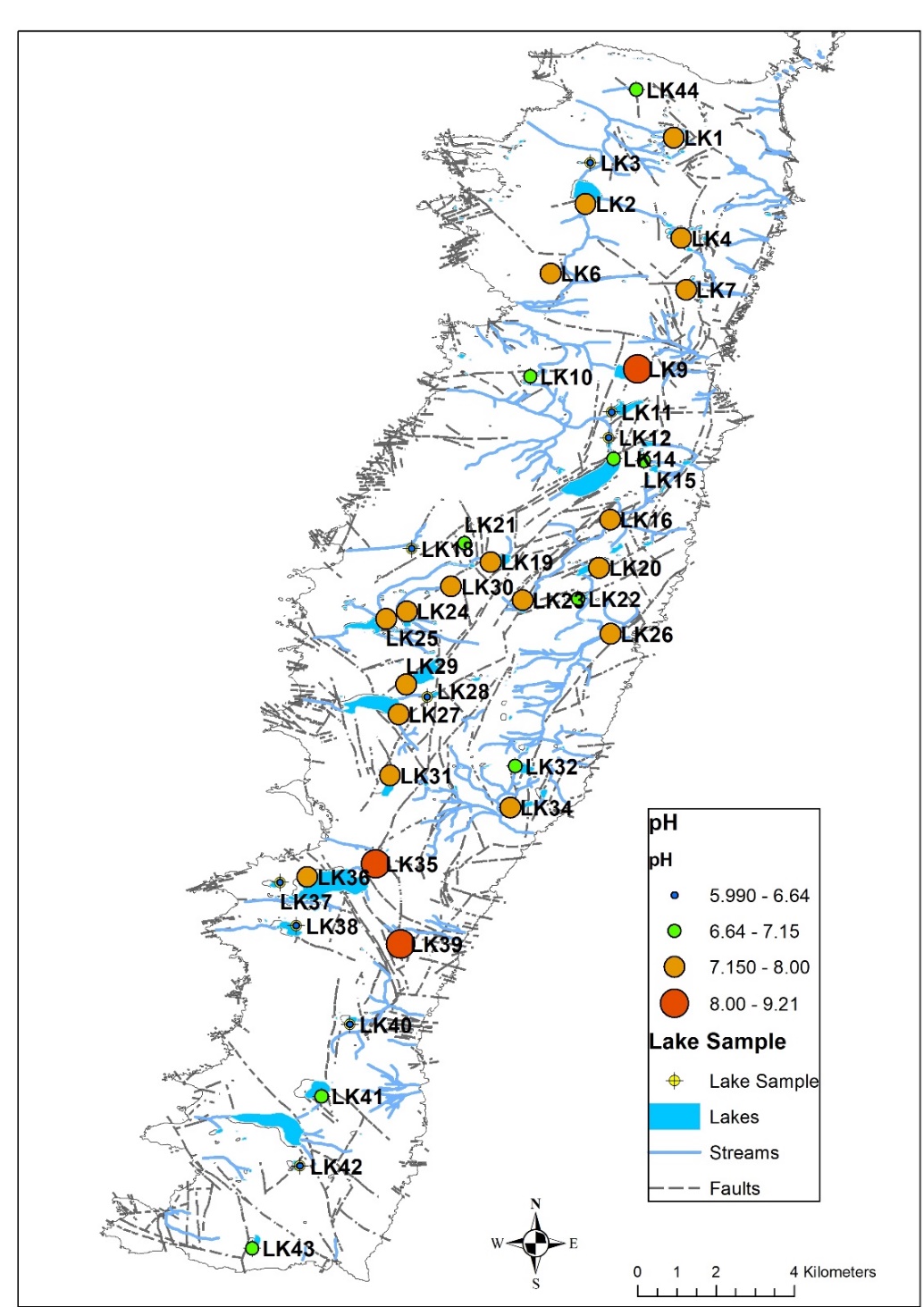

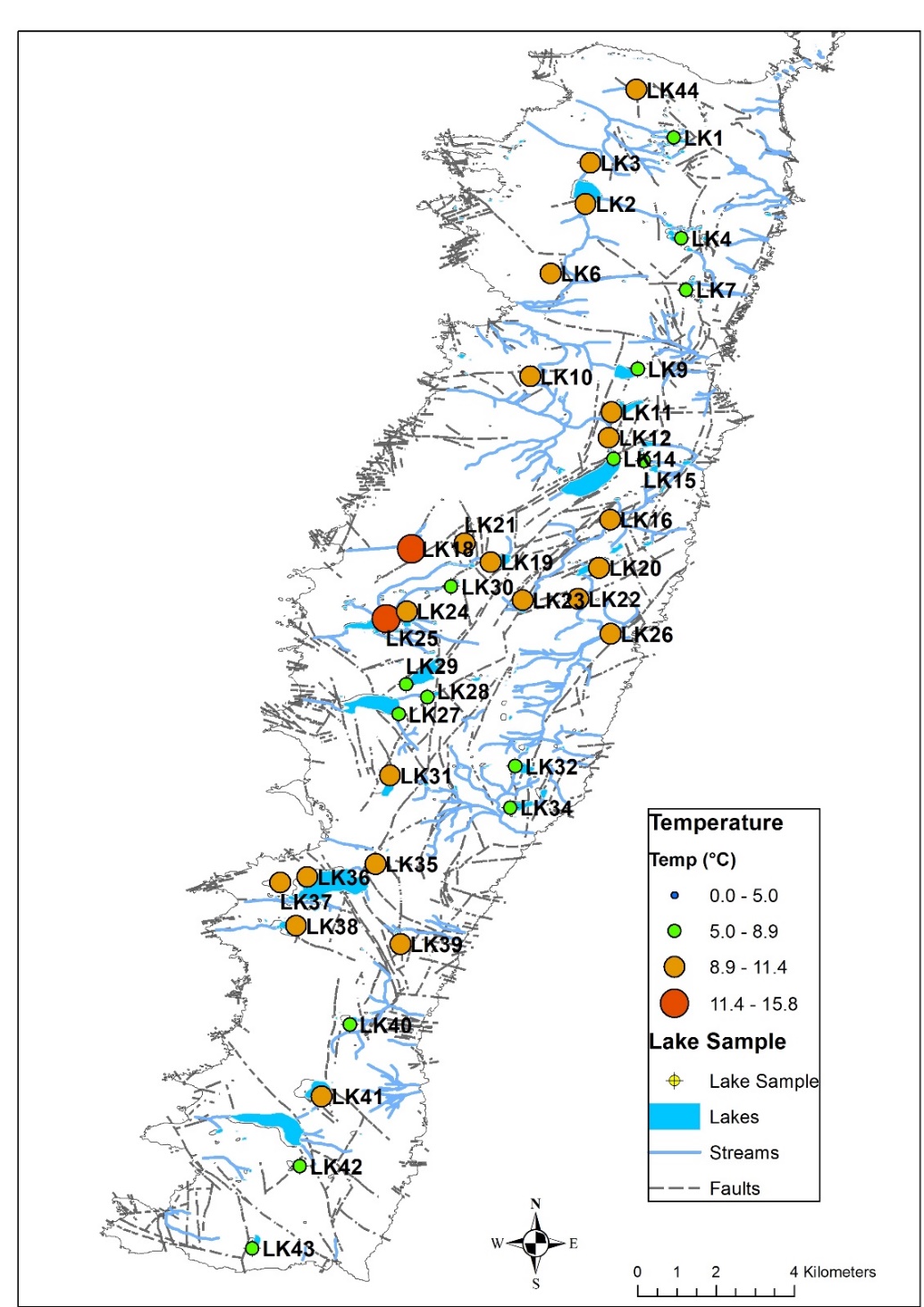


**Fig. S10 pH and temperature at each sampling site. Samples where data is not available are shown as a yellow bullseye symbol. Coloured points represent lake sample locations, with point colour and size representing values shown in the legend.**


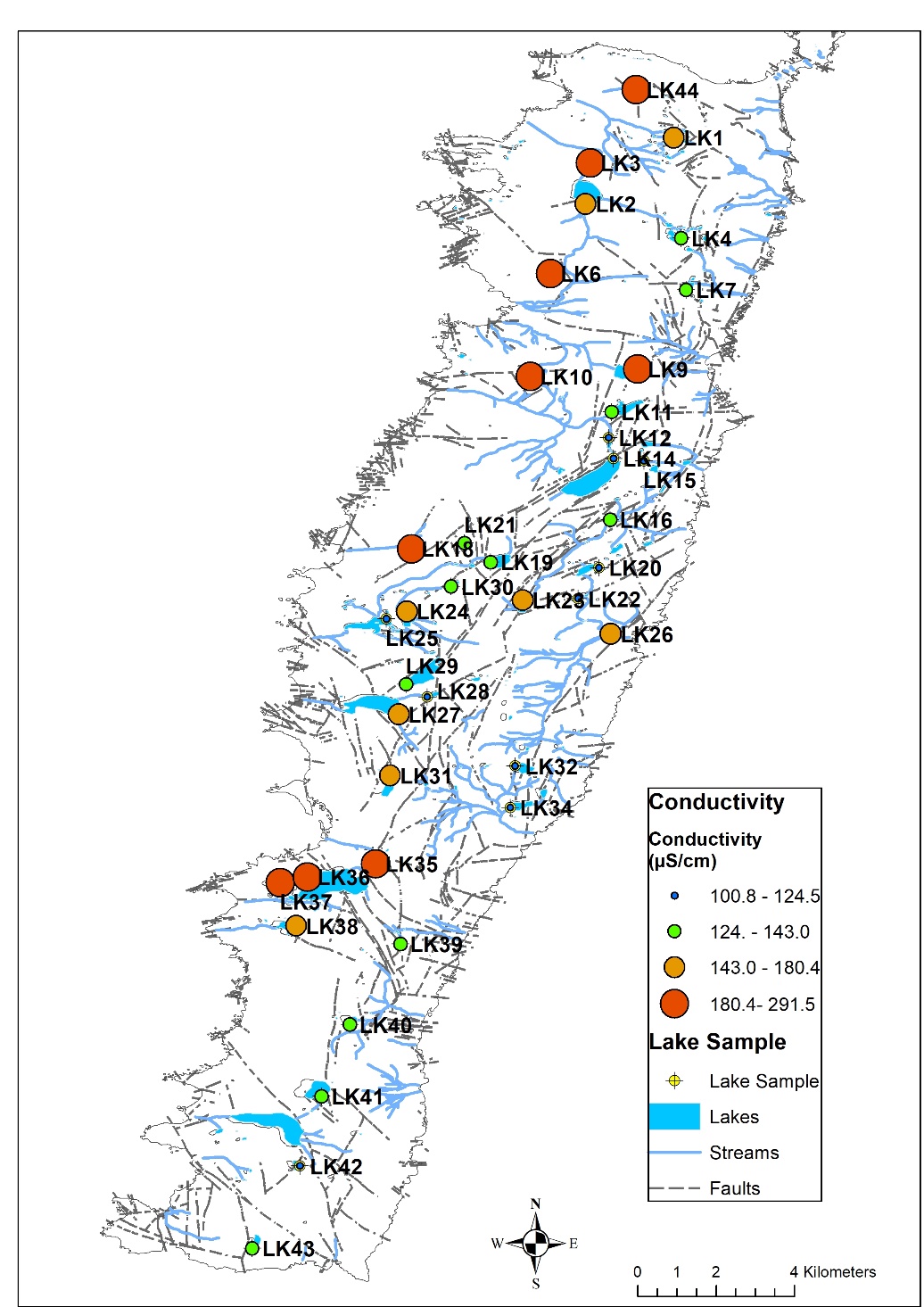

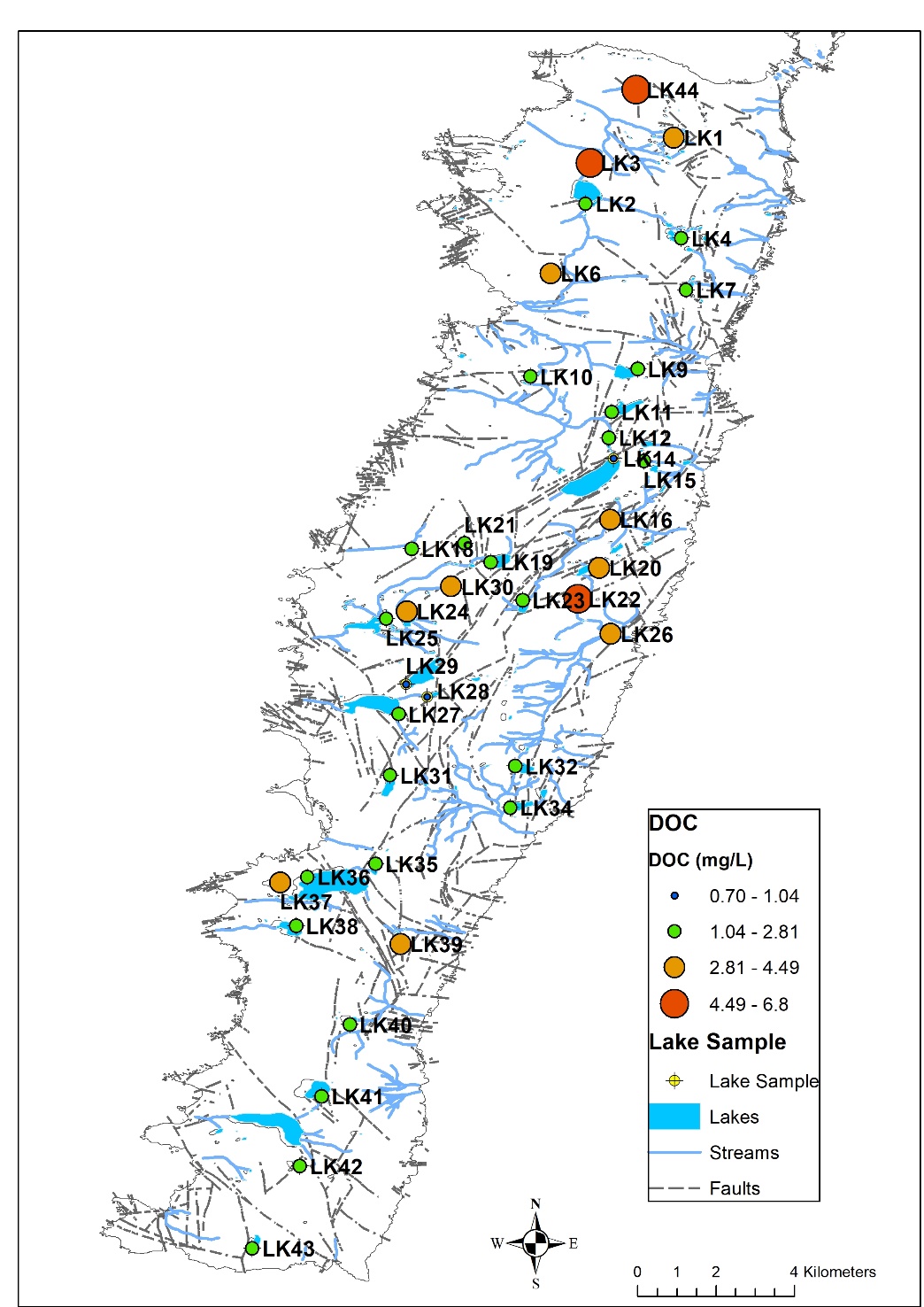


**Fig. S11 Conductivity and dissolved organic carbon (DOC) concentrations at each sampling site. Samples where data is not available are shown as a yellow bullseye symbol. Coloured points represent lake sample locations, with point colour and size representing values shown in the legend.**


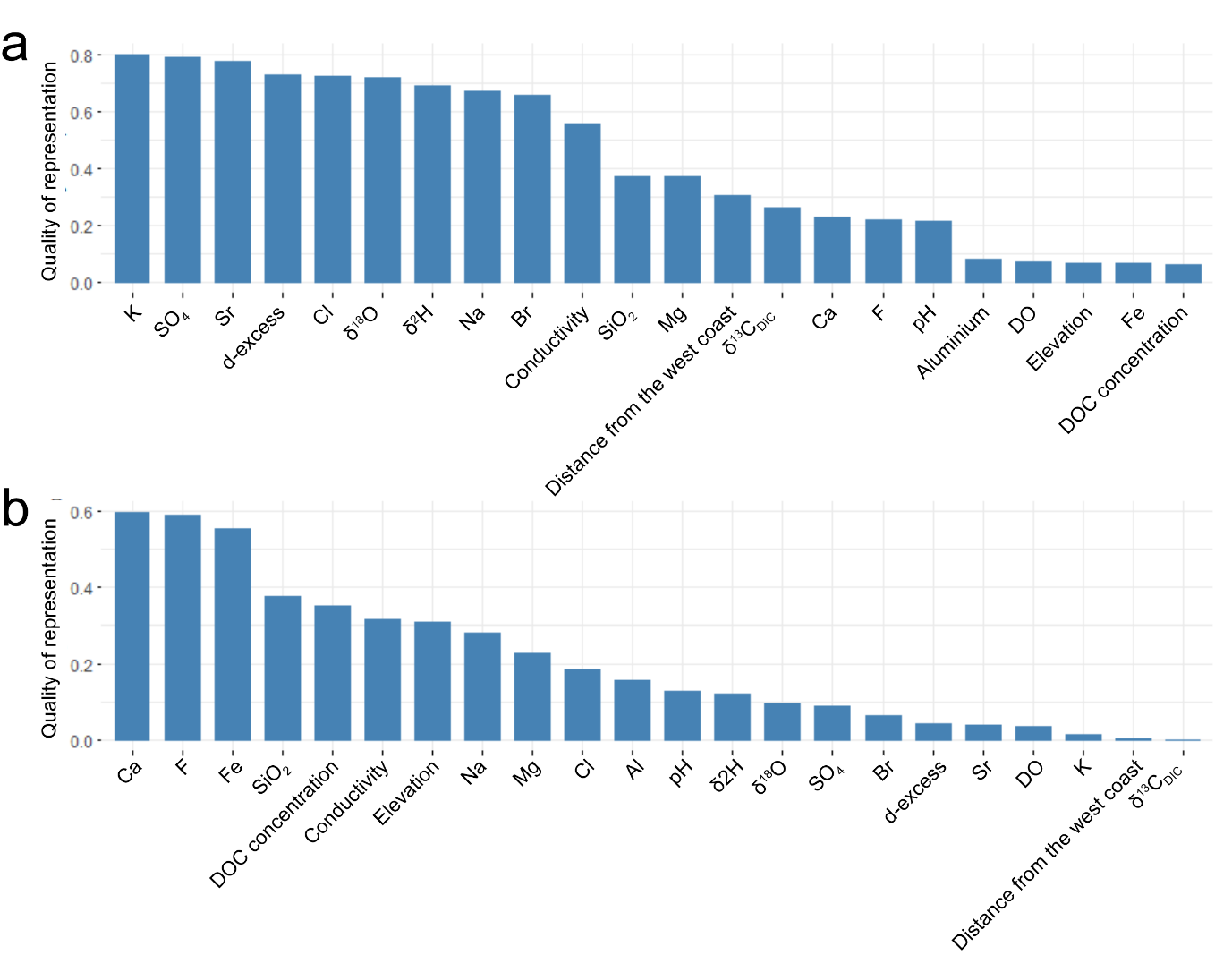


**Fig. S12 PCA results showing variables loading most strongly on (a) component 1 and (b) component 2.**
